# Models of epidemics: when contact repetition and clustering should be included

**DOI:** 10.1186/1742-4682-6-11

**Published:** 2009-06-29

**Authors:** Timo Smieszek, Lena Fiebig, Roland W Scholz

**Affiliations:** 1 Institute for Environmental Decisions, Natural and Social Science Interface, ETH Zurich, Universitaetsstrasse 22, 8092 Zurich, Switzerland; 2 Department of Public Health and Epidemiology, Swiss Tropical Institute, Socinstrasse 57, 4051 Basel, Switzerland

## Abstract

**Background:**

The spread of infectious disease is determined by biological factors, e.g. the duration of the infectious period, and social factors, e.g. the arrangement of potentially contagious contacts. Repetitiveness and clustering of contacts are known to be relevant factors influencing the transmission of droplet or contact transmitted diseases. However, we do not yet completely know under what conditions repetitiveness and clustering should be included for realistically modelling disease spread.

**Methods:**

We compare two different types of individual-based models: One assumes random mixing without repetition of contacts, whereas the other assumes that the same contacts repeat day-by-day. The latter exists in two variants, with and without clustering. We systematically test and compare how the total size of an outbreak differs between these model types depending on the key parameters transmission probability, number of contacts per day, duration of the infectious period, different levels of clustering and varying proportions of repetitive contacts.

**Results:**

The simulation runs under different parameter constellations provide the following results: The difference between both model types is highest for low numbers of contacts per day and low transmission probabilities. The number of contacts and the transmission probability have a higher influence on this difference than the duration of the infectious period. Even when only minor parts of the daily contacts are repetitive and clustered can there be relevant differences compared to a purely random mixing model.

**Conclusion:**

We show that random mixing models provide acceptable estimates of the total outbreak size if the number of contacts per day is high or if the per-contact transmission probability is high, as seen in typical childhood diseases such as measles. In the case of very short infectious periods, for instance, as in Norovirus, models assuming repeating contacts will also behave similarly as random mixing models. If the number of daily contacts or the transmission probability is low, as assumed for MRSA or Ebola, particular consideration should be given to the actual structure of potentially contagious contacts when designing the model.

## Background

The spread of infectious disease is determined by an interplay of biological and social factors [1]. Biological factors are, among others, the virulence of an infectious agent, pre-existing immunity and the pathways of transmission. A major social factor influencing disease spread is the arrangement of potentially contagious contacts between hosts. For instance, the distribution of contacts among the members of a population (degree distribution) strongly impacts population spread patterns: Highly connected individuals become infected very early in the course of an epidemic, while those that are nearly isolated become infected very late, if at all [2,3]. For a high dispersion of the degree distribution, the transmission probability above which diseases spread is lower than for a low dispersion [2-4]. If the degree distribution follows a power law, the transmission probability necessary to sustain a disease even tends to zero [5-7].

Another important structural property influencing the spread of diseases is the clustering of contacts. Clustering deals with how many of an individual's contacts also have contact among each other. High clustering of contacts means more local spread (within cliques) and thus a rapid local depletion of susceptible individuals. In extreme cases, infections get trapped within highly cohesive clusters. Random mixing is known to overestimate the size of an outbreak [8], whereas the local depletion caused by clustering remarkably lowers the rates of disease spread [9,10]: Clustering results in polynomial instead of exponential growth, which can be expected for unclustered contact structures [11].

For most of the diseases transmitted by droplet particles or through close physical contact, the number of contacts that can be realistically made within the infectious period has a clear upper limit. The mean value of potentially contagious contacts can be interpreted in a meaningful way, since the distribution of daily contacts is unimodal with a clear "typical" number of contacts [12-15]. Potentially dominant properties of the underlying contact structure are the clustering of such contacts and their repetitiveness, i.e. whether contacts repeat within the infectious period or not.

A recent study combining a survey and modelling showed that the repetition of contacts plays a relevant role in the spread of diseases transmitted via close physical contact. Contrarily, the impact of repetitiveness seems to be negligible in case of conversational contacts [16]. However, the generality of these findings is limited, as they are based on a small, unrepresentative sample and as the specific patterns of such contacts vary depending on the national and cultural context [12]. A more theoretical work showed that the dampening effect of contact repetition is further increased by contact clustering and is more pronounced if the number of contacts per day is low [10].

The aim of this paper is to better understand the conditions under which the inclusion of contact repetition and clustering is relevant in models of disease spread compared to a reference case assuming random mixing. This is pertinent, as many researchers still use the random mixing assumption without thoroughly discussing its adequacy for the respective case study [17-21]. In particular, we test and discuss the influence of transmission probability, number of contacts per day, duration of the infectious period, clustering and proportion of repetitive contacts on the total outbreak size of a disease. This helps modellers and epidemiologists make informed decisions on whether the simplifying random mixing assumption provides adequate results for a particular public health problem.

## Methods

### Stochastic SIR models

**Figure 1:**
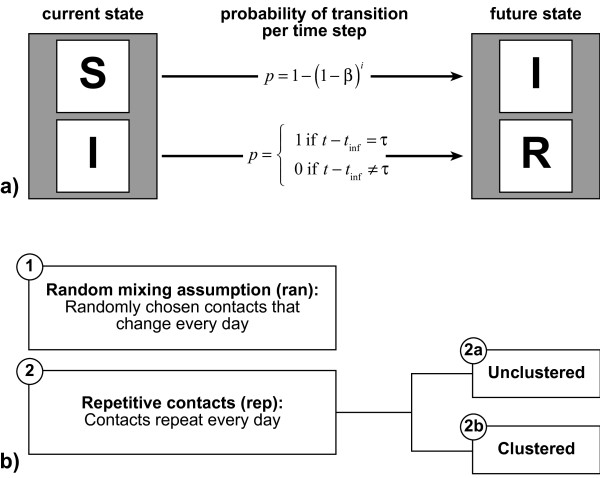
**State transitions and contact structures**. Subfigure a: Two transitions are allowed between three different states an individual can take: (S)usceptible to (I)nfectious and (I)nfectious to (R)ecovered. *β *denotes the transmission probability of one susceptible-infectious pair per time step. *i *stands for the number of infectious contacts that a specific susceptible individual has at the current time step. *t *gives the current simulation time, whereas *t*_inf _gives the time step at which the individual was infected. *τ *is the infectiousperiod. Subfigure b: We compare two model types: the contacts in the first type change daily while those in the second type are constant over time. The second model type assuming repetitive contacts exists in the two variants 2a and 2b.

We assess the influence of repetitive contacts and clustering on the total outbreak size *I*_*tot *_(number of new infections over simulation time) for a simple SIR structure [3,22] under which every individual is either fully susceptible or infectious or recovered (= immune) (cf. figure 1a). We construct two different types of individual-based models: one assuming random mixing (i.e. contacts are unique and not clustered), the other assuming complete contact repetitiveness (i.e. the set of contacts of a specific individual is identical for every simulation day) and allowing for clustering (cf. figure 1b and additional file 1). Both model types can be blended in varying proportions. In our models, every infectious individual infects susceptible contacts at a daily probability *β*, which is equal for all infectious-susceptible pairs. Individuals remain infectious for an infectious period *τ*, which is exactly defined and not stochastic in its duration. Infectious individuals turn into the recovered state as soon as the infectious period passed by. We assume that infection confers full immunity for the time scale of the simulation. Hence, recovered individuals cannot be reinfected by further contacts with infectious persons. There are no birth or death processes: Hence, the population size is constant. All possible state transitions are delineated in figure 1a.

Under the random mixing assumption (in mathematical terms denoted by index *ran*), *n *contacts are randomly chosen out of the whole population (including susceptible, infectious and recovered individuals) for every individual and every day. There is neither contact repetition nor clustering, as our algorithm ensures, that no contact partner is picked twice by the same individual.

In fact, clustering is neither properly defined nor is it a reasonable concept under the random mixing assumption for theoretical and practical reasons: In this paper we refer to the common definition that the clustering coefficient *CC *is the ratio of closed triplets to possible triplets [23], where a closed triplet is defined as three individuals with mutual contact. This definition is based on static networks. As in random mixing models contacts change daily, different clustering coefficients could be calculated for every single simulation time step. However, no epidemiologically relevant effect of such clusters could be observed, because any new infection comes into effect only in the following time step when contacts are already rearranged. As a consequence, there is no local depletion of susceptible individuals observable under this definition, even for high clustering coefficients. If clustering would be defined for an extended time interval (e.g., the infectious period), an enormous amount of closed triplets would be necessary to attain only slight clustering coefficients as the total number of contacts over such a long time is very high. For such huge cliques, there is no meaningful interpretation and no analogy in the real world.

Repetitive contacts (in mathematical terms denoted by index *rep*) are implemented by generating a static network with *n *links for every individual. The links of this network represent stable, mutual, daily contacts between individuals. As mentioned, the model type assuming repetitive contacts exists in two variants. For the variant without clustering, individuals are linked completely at random. Nonetheless, for repetitive contacts, clustering is a meaningful concept as contacts are static and as clusters correspond to observable entities in the real world: Family or work contacts, for instance, are usually clustered and tend to be highly repetitive. In this paper, predefined average clustering coefficients are achieved by alternately generating random links and triplet closures, as suggested by Eames [10], until the clustering aim is achieved in average for the whole population. When the target value of closed triplets is reached, the network is filled up with random contacts until all individuals have *n *contacts.

This paper compares most parameter settings for a model assuming either full random mixing or perfect repetitiveness of contacts. This comparison allows for estimating the maximal possible difference between both antipodal simplifications of reality. However, real world dynamics of networks are far more complicated; therein some contacts are repeated daily, others on certain days of the week and others only once in a while. In order to investigate the effect of different proportions of repetitive contacts, we vary the fractions of repetitive contacts.

### Parameter space to be tested

In the following section, we describe some important factors in the spread of infectious diseases that will be systematically tested for their influence on the difference between the random mixing model and the model assuming repetitiveness (with and without clustering).

Important biological factors influencing the spread of infectious diseases are the duration of the infectious period *τ *and the per-contact transmission probability *β*.

The *infectious period τ *stands for the number of days (simulation time steps) a newly infected individual will remain infectious. The effect of repetitive contacts is tested for diseases with *τ *values between 2 and 14 days (see *τ *values given for various diseases in table 1).

**Table 1:** Key transmission parameters of selected diseases

**Disease**	*R*_0_	*τ ***[d]**	**Transmission pathways **[32]
Chickenpox (Varicella)	7–12[3]	10–11[3]	Direct contact, airborne, droplet, contact with infectious material

Ebola	1.34[42]^a^ 1.79[43] 1.83[42]^b^ 2.13[43]^c, a^ 3.07[43]^c, b^	14[43]	Direct contact, contact with infectious material, monkey-to-person

Influenza	1.3; 1.8; 3.1[17]^d^ 1.39[51] 1.58; 2.52; 3.41[52]^e^ 1.7–2.0[53] 2–3[54]^f^ 3.77[55]	2–3[3] 2.27[55] 3–7[56]	Direct contact, airborne, droplet [57]

Measles	5–18[3] 7.17–45.41[33]^g, h^ 7.7[34] 15–17[32] 16.32[33] g	6–7[3]	Direct contact, airborne, droplet, contact with infectious secretions

MRSA^i^	1.2[41]^j^	as long as purulent lesions continue to drain[40]	Direct contact, contact with infectious material[40]

Mumps	7–14[3] 4.4[35]^h^ 10–12[32]	4–8[3]	Direct contact, airborne, droplet, contact with infectious secretions

Norovirus	3.74[37]^j^	1.8[37]^j^	Direct contact, droplet (vomiting), contaminated food[38,39]^k^

SARS^k^	1.43[43]^l^ 1.5[43]^m^ 1.6[47] 2.2–3.7[48] >2.37[49]	4[49] 5[43]	Close direct contact

Whooping cough (Pertussis)	10–18[3] 15–17[32]	7–10 [3]	Direct contact, airborne, droplet, contact with infectious secretion

Abbreviations, data sources and methods for the calculation of *R*_0_, as far as known: ^a ^outbreak Uganda 2000 [44]; ^b ^outbreak Congo 1995 [45]; ^c ^regression estimates; ^d ^1918 pandemic data from an institutional setting in New Zealand [17]; ^e ^1918 pandemic data from Prussia; assuming serial intervals of 1, 3 and 5 days [52]; ^f ^1918 pandemic data from 45 cities of the United States [54]; ^g ^data from six Western European countries [33]; ^h ^age structured homogenous mixing model; ^i ^MRSA, Methicillin-Resistant *Staphylococcus Aureus*;^j ^hospital outbreaks; ^k ^SARS, Severe Acute Respiratory Syndrome;^l ^outbreak Singapore 2003 [50]; ^m ^outbreak Hong Kong 2003 [50]

The *transmission probability β *is defined as the probability that an infectious-susceptible pair results in disease transmission within one single time step of the simulation. *β *is equal for every infectious-susceptible pair. The effect of *β *on the impact of repetitive contacts compared to the reference case (without repetitive contacts) is analyzed via systematic variation.

In the results section, we show all results for *β*·*n*·*τ *values instead of pure *β *values to assure comparability of the outcomes: *β*·*n*·*τ *equals the basic reproduction number *R*_0 _for the random mixing model and thus models with the same *β*·*n*·*τ *result in a similar total outbreak size. Referring to *β*·*n*·*τ *values assures that model comparisons are always made for a relevant range of *β*. The effect of repetitive contacts is tested for *β*·*n*·*τ *values between 1.2 and 4.0 in increments of 0.2. The epidemic threshold of random mixing models is *β*·*n*·*τ *= 1.0. As we are only interested in diseases that can cause an epidemic, we set the lower boundary to 1.2. The upper boundary is chosen arbitrarily.

Social factors considered in this paper are the number of contacts per day *n*, the proportion of repetitive contacts and the clustering coefficient.

For every single simulation run, the *number of contacts per day n *is constant and equal for all individuals. *n *counts every contact an individual has within one simulation step, regardless of the alter's infection status (susceptible, infectious or recovered) and regardless of whether the contact is repetitive. The effect of repetitive contacts on the simulation outcome is tested for *n *values between 4 and 20 with a step width of 2 (mean values for conversational contacts lie in this range [12]).

In order to investigate the effect of varying *fractions of repetitive contacts*, we simulate the total outbreak size for 0%, 25%, 50%, 75% and 100% repetitive contacts. Thereby, 25% repetitive contacts means that one fourth of all contacts on a given day repeat daily but that three fourth of the contacts on a given day are unique.

In the case of repetitive contacts, *clustering coefficients *between *CC *= 0.0 and 0.6 with a step width of 0.2 are accounted for. This span covers a wide range of existing transmission systems from highly infectious diseases with a high number of contacts per day and with clustering coefficients close to zero to highly structured settings with a considerable proportion of clustered contacts like in hospitals [24].

For all runs of the simulation model, the total population *N *was fixed to 20000 individuals. As initial seed 15 randomly chosen individuals are set to infectious every simulation run. For each combination of model parameters 350 runs were performed to achieve stable mean values of the outcome variables. A simulation run was terminated when no infectious individual was left.

### Overview on performed analyses

We test the influence of the abovementioned parameters on the difference between the model typed in three distinct analyses. First, we show how strongly the total outbreak sizes *I*_*tot*, *ram *_and *I*_*tot*, *rep *_differ depending on *τ*, *n *and *β*. In the second analysis we vary *n *and *β *and the clustering coefficient *CC *for the case of repetitive contacts. Thirdly, we show how the total outbreak size changes under various *n*, *β *and *CC*, when repetitive and random contacts are mixed in varying proportions. Details for the three analyses are given in table 2.

**Table 2:** Parameter settings of the analyses

	*n*	*τ ***[d]**	*β*·*n*·*τ*	*CC*	**Proportion repetitive contacts**
Analysis 1					
a	4 – 20; 2	2 – 14; 1	1.6	.0	.0 vs. 1.0
b	4 – 20; 2	14	1.2 – 4.0; .2	.0	.0 vs. 1.0
c	4	2 – 14; 1	1.2 – 4.0; .2	.0	.0 vs. 1.0
Analysis 2	4 – 20; 2	14	1.2 – 4.0; .2	.0 – .6; .2	.0 vs. 1.0
Analysis 3	8 – 20; 4	14	1.2 – 3.0; .6	.0 – .6; .2	.0 – 1.0; .25

Parameter ranges are given before the semicolon; the increment is given after the semicolon. Single numbers stand for fixed values.

In addition to the total outbreak size, we present further epidemiologically relevant indicators in the additional files. Epidemic curves can be found in additional file 2, findings on the model differences regarding the average peak size of the outbreaks and the average time to peak are given in additional file 3.

## Results and discussion

### Analysis 1: The effect of contact repetition depending on *τ*, *n *and *β*

**Figure 2:**
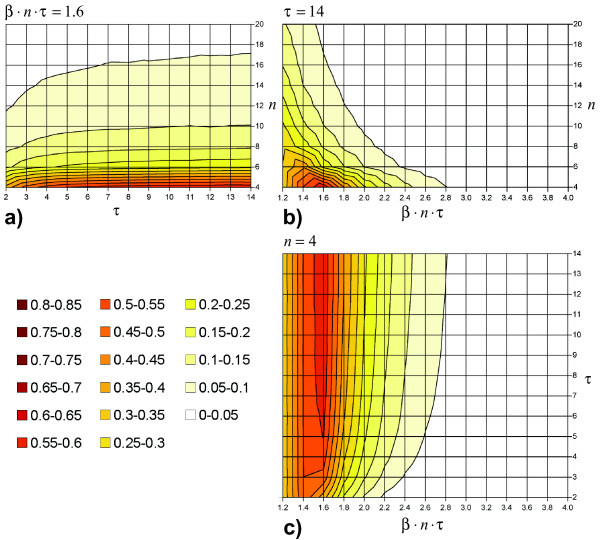
**Model differences depending on *τ*, *n *and *β***. Subfigures a-c show the difference in the total outbreak size between a pure random mixing model and a model assuming complete repetitiveness (without clustering) relative to the population size *N*. Contour plots are interpolated from a grid of measurement points using Microsoft^® ^Office Excel 2003. (a) infectious period: 2 ≤ *τ *≤ 14, step width (sw): sw = 1; daily number of contacts: 4 ≤ *n *≤ 20, sw = 2; per-contact transmission probability: *β*·*n*·*τ *= 1.6. (b) 1.2 ≤ *β*·*n*·*τ *≤ 4.0, sw = .2; 4 ≤ *n *≤ 20, sw = 2; *τ *= 14. (c) 1.2 ≤ *β*·*n*·*τ *≤ 4.0, sw = .2; 2 ≤ *τ *≤ 14, sw = 1; *n *= 4.

As described in the methods section, *τ*, *n *and *β*·*n*·*τ *have been varied systematically to investigate the difference between the mean values of the outbreak sizes  and  under different parameter constellations. Figures 2a–c show three contour plots in which the difference between both model types  is given for various *τ*, *n *and *β *values. Figure 2a gives  depending on 4 ≤ *n *≤ 20 and 2 ≤ *τ *≤ 14 with a fixed *β*·*n*·*τ *= 1.6. The total outbreak size depends strongly on the number of contacts per day *n *but only slightly on the infectious period *τ*. In case of an infectious period between two and four days, there is a considerable change of  with Δ*τ*; for 4 <*τ *≤ 8, slight changes are observable; in case of infectious periods over eight days, the difference between both models depends mainly on *n*. Figure 2b gives  depending on 4 ≤ *n *≤ 20 and 1.2 ≤ *β*·*n*·*τ *≤ 4.0 with a fixed *τ *= 14. It shows that the difference between both models depends strongly on both parameters, the number of daily contacts *n *and the transmission probability *β*. Differences are large for a small *n *or small *β *but negligible for a large *n *when *β *is large at the same time. Figure 2c, showing  for 1.2 ≤ *β*·*n*·*τ *≤ 4.0, 2 ≤ *τ *≤ 14 and *n *= 4, is consistent with the observations made for the other two figures.

#### Effect of contact number

The increasing difference between  and  with decreasing *n *can be explained by two lines of reasoning.

First, in the case of contact repetition, there is always at least one out of the *n *contacts per day that is already infected (and thus not available for new infection): As contacts are stable over time, the infector of a susceptible individual is included in the subsequent contact list of that individual even when said individual has changed to the infectious state. Thus, at the least, the contact that originally transmitted the infection is not susceptible. In contrast, contacts change in every time step under the random mixing assumption: Hence, the infector is not more likely to appear in the contact set than any other individual. This difference between  and  is more pronounced for small *n *because one non-susceptible individual out of a small set of contacts means a relatively higher decrease in local resources than does one out of a large set of contacts.

Secondly, any new infection means that the infector will have one susceptible contact less for all subsequent time steps. This local depletion of resources is more pronounced for small *n *for the same reason as in the first argument. Further, stochasticity acts stronger in small local environments than in large ones [25].

**Figure 3:**
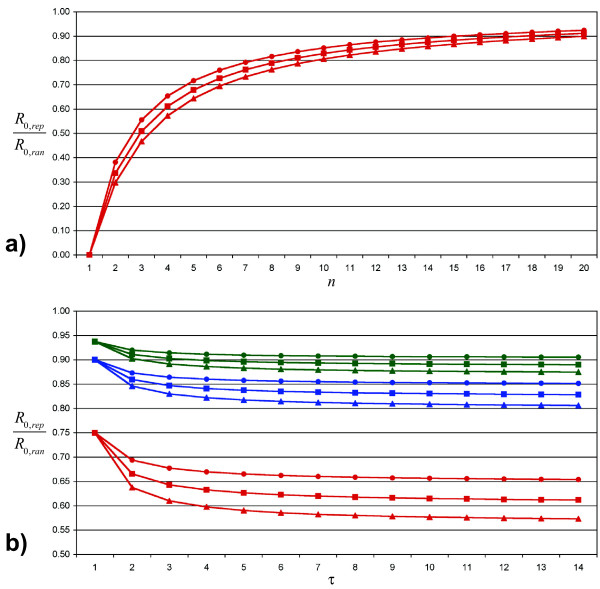
**Ratio of the basic reproduction numbers**. Subfigure a shows the ratio *R*_0,*rep*_/*R*_0,*ran *_(as defined in equation 1) for 1 ≤ *n *≤ 20 (number of daily contacts) and *τ *= 14 (infectious period). Triangles stand for *β*·*n*·*τ *= *R*_0,*ran *_= 2.4, squares for *R*_0,*ran *_= 1.8 and circles for *R*_0,*ran *_= 1.2. Subfigure b gives *R*_0,*rep*_/*R*_0,*ran *_depending on the infectious period *τ*. Red lines and symbols are for *n *= 4, and blue lines stand for *n *= 10, whereas green lines represent *n *= 16. The meaning of the symbols is identical as in subfigure a.

Both effects can also be seen in the equation 1, which gives *R*_0,*rep *_as a function of *R*_0,*ran*_, *n *and *τ *(see also figure 3a; details for equation 1 are given in additional file 4):

(1)

In this equation the number of susceptible individuals in the local environment is reduced by 1 compared to the random mixing case, as we assume that every contact except the one that originally transmitted the infection is susceptible. This number of susceptible individuals (*n *- 1) is multiplied by the probability that such an individual becomes infected during the infectious period *τ*. As (*n *- 1) is smaller than *n *and [1 - (1 - *β*)^*τ*^] is smaller (or equal for *τ *= 1) than *β*·*τ*, the expected number of secondary cases caused by an infectious individual in a population with a huge number of susceptible and few infected ones is always smaller in the repetitive case.

#### Effect of the per-contact transmission probability

The difference between  and  decreases rapidly with increasing *β*. The reason is that practically every individual will be reached and infected in case of large transmission probabilities, regardless of the underlying contact structure. Differences between both models may appear in the shape of the outbreak curve (cf. to additional files 2 and 3), but in terms of *I*_*tot *_both models are equivalent. In case of small transmission probabilities, differences in the effective number of secondary cases generated by an infectious individual can become visible, as only a fraction of the whole population will be infected under both assumptions.

#### Effect of the infectious period

As expected, the difference between  and  increases with increasing *τ*. However, the change in difference is largest for Δ*τ *in a range of low *τ *values, but is almost irrelevant for high values of *τ*. This observation is explained by the *τ*-dependence of *R*_0,*rep *_(equation 1, see also figure 3b): The longer the infectious period, the smaller the chances for a specific contact to remain uninfected. However, this increase in individual infection probability is partly compensated by a lower per-day transmission probability, which is needed to achieve constant *R*_0,*ran*_. The interaction of these antagonistic effects results in a stabilization of *R*_0,*rep*_/*R*_0,*ran *_for a large *τ*.

### Analysis 2: The effect of contact repetition combined with clustering depending on *n *and *β*

**Figure 4:**
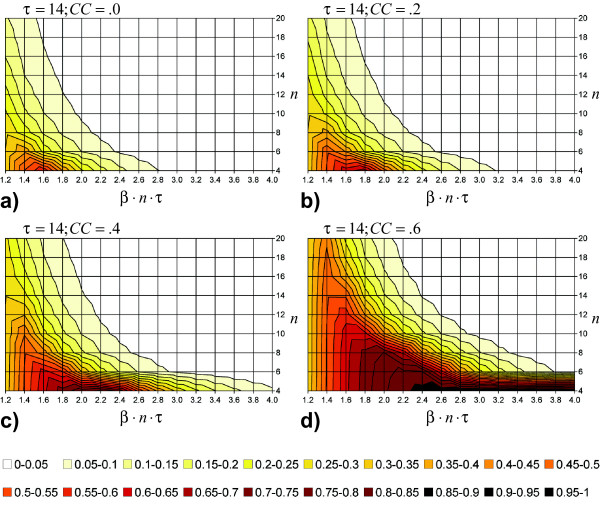
**Dampening effect of clustering**. Subfigures a-d show the difference in the total outbreak size between a pure random mixing model and a model assuming complete repetitiveness (with different levels of clustering) relative to the population size *N *for 4 ≤ *n *≤ 20, 1.2 ≤ *β*·*n*·*τ *≤ 4.0 and *τ *= 14. Subfigure 4a is identical with subfigure 2b. The clustering coefficient *CC *is increased picture-wise in steps of .2.

The results presented previously show that  depends mainly on *n *and *β*. In a second step, we investigate how the difference between model type 1 and 2 changes, if clustering is introduced in the latter. Figures 4a–d show the difference between both model types for clustering coefficients *CC *between 0.0 and 0.6 when *τ *is fixed to 14 days and when *n *and *β*·*n*·*τ *vary in the ranges mentioned above. As expected, clustering results in an increased difference between both model assumptions. This increase is most pronounced for small numbers of contacts per day. The peak of  is constantly at *n *= 4 but shows a right shift on the *β*·*n*·*τ *axis for increasing *CC*.

The further dampening of disease spread by clustering can be explained by increased locality of resources: While repetition limits the number of available susceptible individuals by keeping previously infected ones in the set of contacts, clustering reduces the number of susceptible contacts because there is a higher likelihood that contacts of an infector have already become infected by others during the infectious period, as infections spread rapidly within cliques. The reason why this effect is more pronounced for small *n *rather than for large *n *is the same as in the case of unclustered, pure contact repetition: Any reduction of susceptible individuals in the set of contacts weights relatively stronger in the case of few contacts than in the case of many. The right shift of the peak of  can be explained by the increased transmission probability *β *needed to pass the epidemic threshold under increased clustering compared to the constantly low levels of *β *necessary under the random mixing assumption [26].

### Analysis 3: Varying proportions of contact repetition, clustering and *β*

**Figure 5:**
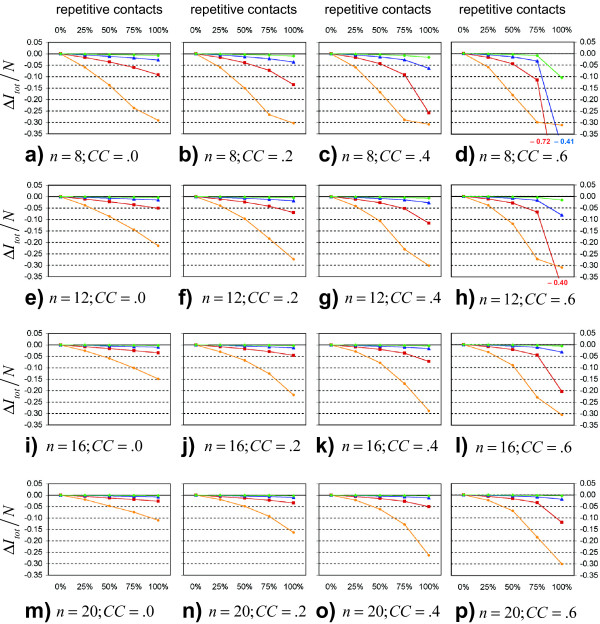
**Mixed models**. Subfigures a-p show the decrease of the total outbreak size relative to the size of the total population when the fraction of repetitive and clustered contacts is increased. 25% rep means that one fourth of all contacts on a given day repeat every day but that three fourths of the contacts on a given day are unique. Clustering coefficients *CC *are only defined and calculated for the repetitive fraction of the contacts. All simulations were calculated for an infectious period of 14 days. Orange circles stand for *β*·*n*·*τ *= 1.2, red squares for *β*·*n*·*τ *= 1.8, blue triangles for *β*·*n*·*τ *= 2.4 and green rhombi for *β*·*n*·*τ *= 3.0. The number of daily contacts *n *increases in steps of 4 per line of the subfigures, beginning with *n *= 8 in the first line. The first column of the subfigures shows *CC *= .0, the second column *CC *= .2, the third column *CC *= .4 and the fourth column *CC *= .6.

We simulated the difference between both model assumptions for all possible combinations of *n *= 8, 12, 16 and 20, *β*· *n*·*τ *= 1.2, 1.8, 2.4 and 3.0, *τ *= 14 and *CC *= 0.0, 0.2, 0.4 and 0.6. The simulation results are shown in figures 5a–p. The relation between the proportion of repetitive contacts per day and the average difference between this mixed model and a model assuming purely random mixing is approximately linear in the absence of clustering (for all tested cases, linear regressions between the proportion of repetitive contacts per day and the deviation of  from the purely random mixing model achieve *R*^2 ^> .98). However, the deviation from the random mixing model increases disproportionately with the fraction of repetitive contacts when clustering is introduced (cf. to figures 5b–d, f–h, j–l and 5n–p).

One mechanism driving this non-linear relation when clustering is present is the local depletion of resources. Repetitive contacts of an infector have a much higher chance of becoming infected than do non-repetitive contacts. Moreover, if these repetitive contacts are also highly clustered, it is likely that the disease will become trapped in those cohesive social subgroups. However, if only a few non-repetitive, non-clustered contacts are added per day, the chances of spreading the disease between otherwise unrelated regions of the social network greatly increase.

### Limitations

This paper systematically investigates a variety of epidemiologically relevant parameters needed to describe real-world transmission systems of diseases spread by droplet particles or direct physical contact. However, real-world social and biological processes involved in the transmission of infectious diseases are far more complex than captured by the archetypical model structures presented. Conceptual decisions and simplifications which could have potentially influenced the results are critically discussed in the following:

#### Model structure

We designed our two model types as SIR models, assuming that every individual is either susceptible, infectious or immune with respect to a certain disease. Transitions are only allowed from susceptible to infectious or from infectious to immune. The SIR structure is a fairly good representation for many diseases which lead to full immunity after recovery (e.g., measles). However, many diseases require other representations, as relevant intermediate states need to be covered, e.g., as with a long latency period in SEIR (Susceptible-Exposed-Infectious-Recovered) models. Another common deviation from the SIR structure arises, when recovery confers only partial or no immunity. In such cases, SIS (Susceptible-Infectious-Susceptible) representations are often chosen. In SIR or SEIR models, a total outbreak size can be defined (because the disease fades out at the end of an epidemic), whereas SIS models typically achieve an equilibrium *I*(*t*) in the long run, but the disease does not die out. Despite all the differences in model behaviour, we expect the rough picture to be the same for SIR, SEIR and SIS models, as the mechanisms behind the observed differences for SIR models that we discussed also apply to SIS and SEIR models. Thus, the general conclusions derived in this paper should also hold true for these model types.

#### Degree distribution

The number of daily contacts *n *is fixed and equal for the entire population in both modelling approaches presented. This is a reasonable simplification for the purpose of this paper, as it keeps the investigated number of interactions manageable. However, in real world systems, the number of daily contacts appears to follow a negative binomial distribution [12,14] with some people having a relatively high number of contacts and others being almost isolated. It is known that the variance of the degree distribution impacts the spread of infectious disease, for instance, by decreasing the transmission probability needed to cause an epidemic [27]. Particularly relevant for the difference between random mixing models and models accounting for contact repetition and clustering are the correlations between the number of contacts per day and contact repetition and clustering, respectively. It is plausible to assume that individuals with many contacts tend to also have many unrepeated contacts, whereas individuals with few contacts tend to have disproportionately high levels of repetitive contacts. If the proportion of repetitive contacts and clustering is correlated with the number of contacts, individuals with few contacts are likely to be dead-end streets for infectious diseases. In contrast, highly connected individuals could be structurally more important than expected, as they bridge distinct cliques.

#### Occasional contact repetition

In our simulations, contacts repeat either daily or never. Intermediate states between both extremes of complete random mixing and complete contact repetition have been investigated by combining both models in defined proportions. However, in reality, specific persons can be met at any frequency between never and daily. It is plausible to assume that intermediate frequencies reduce the effect of repetitiveness depending on the duration of the infectious period *τ*: For short infectious periods, those with low contact frequencies might appear as unrepeated contacts whereas they unfold their full dampening potential for long infectious periods.

#### Contact intensity and duration

In our models all contacts between an infector and a susceptible individual are equally likely to result in the transmission of the infectious disease. This simplification is not a good representation of the real world: The transmission probability depends on the amount of infectious material ingested by a susceptible person [28,29]. The uptake correlates with contact duration and intensity. Contact duration is long for highly repetitive contacts, while unrepeated contacts tend to have short duration (unpublished data). Accordingly, it can be expected that the interaction of clustering, contact repetitiveness and contact duration leads to a rapid infection of all closely tied clusters (primarily families, then workgroups and cliques at school and childcare institutions), leaving behind the people connected via mainly short, unclustered, occasional contacts.

#### Distribution of infectious period

The infectious period *τ *is fixed in our model, which contrasts to the design of classical mean-field models assuming exponentially distributed infectious periods [3,22]. Keeling and Grenfell argue that *R*_0 _is smaller for exponential period models than for fixed period models under otherwise identical conditions, because individuals with a long *τ *rapidly exhaust the susceptible in their local neighbourhood and, therefore, cannot compensate for the large majority of individuals with extremely short infectious periods [25,30]. However, the often assumed exponential distribution is highly unrealistic, as observed infectious periods tend to be closely centred around a mean period and are thus less dispersed [31]. Thus, assuming a fixed infectious period is a reasonable simplification of the reality that is not likely to have a major influence on  as only very few individuals will use up their local susceptible resources during the infectious period in most cases. Moreover, if the infection probability is high enough to exploit almost the entire local environment (such that deviations of *τ *could affect the individual reproduction ratio),  will reach the order of magnitude of the population size in either the fixed or the exponential case.

### Implications for some exemplar diseases

Information on the per-contact transmission rate *β *and the number of potentially contagious contacts *n *is often not easily accessible or available and has to be measured (or fitted) if included in models of disease spread. However, rough estimates of both variables can be obtained when *R*_0 _estimates are available and when the possible pathways of transmission are known, because *β *and *n *are linked to the basic reproduction number by *R*_0,*ran *_= *β*·*n*·*τ *and the possible pathways reveal information on the possible number and structure of contacts at risk: At one extreme there is transmission via close physical contacts, which correlate mostly with intense social relations and are typically rare, repetitive and highly clustered. The other extreme is airborne transmission via tiny droplet nuclei that remain suspended indoors for a long time. In this case, vast numbers of persons can potentially be exposed, and such casual contacts are neither highly repetitive nor strongly clustered.

Table 1 provides information about the infectious period *τ*, *R*_0 _estimates and the possible pathways of transmission for a variety of infectious diseases. The implications of clustering and contact repetition for models of the diseases listed in this table are discussed below.

Typical childhood diseases like mumps, measles, pertussis (whopping cough) or chickenpox have comparatively high *R*_0 _estimates [3,32-35], which means that one infector generates many secondary cases if a sufficient number of susceptible contact partners are available. These diseases are highly communicable – in fact, measles is one of the most highly communicable diseases in the world [36] – and thus, very short and non-intense contacts have the potential to confer infection. Accordingly, both the number of contacts per day *n *and the per-contact transmission probability *β *are very high. We further assume that a high proportion of the contacts are casual contacts, because the threshold for a contact to be potentially contagious is very low with respect to duration and intensity. Consequently, the levels of repetitiveness and clustering are low, which means that the contact patterns for such childhood diseases are structurally similar to random mixing. Considering that high numbers of daily contacts *n *make both types of models that we discussed behave similarly and considering that under high transmission probabilities *β *almost every individual will be reached, random mixing models achieve almost the same results as more elaborate models including a certain amount of contact repetition and clustering. Also in case of Norovirus, the difference  is probably small, as the infectious period of this infectious agent is very short [37] and as at the same time the basic reproduction number is comparatively high [37] (because the disease is easily communicable [38,39]).

On the other side, there are diseases with comparatively low *R*_0 _estimates and typically low numbers of contacts that still qualify for potential transmission. Methicillin-resistant *Staphylococcus aureus *(MRSA), for instance, is an infectious agent mostly transmitted in health care and nursing institutions. It needs close physical contact for transmission [40] and *R*_0 _estimates given in the literature are close to the epidemic threshold [41]. Accordingly, both *β *and *n *are low. At the same time, health care settings tend to be highly structured regarding who cares for whom and who shares a room with whom. Hence, high levels of contact repetitiveness and clustering can be assumed [24]. Modelling MRSA under the random mixing assumption is likely to overestimate the total number of cases for given *n*, *β *and *τ*. If, in contrast, a random mixing model is fitted to measured data from an outbreak, either the infectivity or the number of potentially infectious contacts will be underestimated to meet the measured outbreak size. A similar argumentation applies to Ebola, which is transmitted via direct contact with infected blood, secretions, organs or semen (thus, *n *is rather low) and seems to be only moderately infectious [42-45]. As a consequence, random mixing models of Ebola [46] are of limited validity.

Finally, there are some diseases not easily attributable to one or the other class. Severe Acute Respiratory Syndrome (SARS) and Influenza, for instance, have a range of *R*_0 _estimates between 1.43 and 3.7 [43,47-50] and between 1.3 and 3.77 [17,51-56], respectively. No definite consensus has been reached on whether Influenza is transmitted predominantly by large droplets and close contact or by very small droplets that disseminate quickly and stay suspended in indoor air for a long time [57]. In the latter case, a large amount of people would be at risk of infection, so random mixing would be a reasonable approximation of the real contact patterns. In the case of transmission by close contact and large droplets (that fall out quickly), the mean number of potentially contagious contacts per day lies between 8 and 18, depending on the national and cultural context [12]. Considering that not all contacts are equally likely to transmit influenza, but that long and intense contacts (such as household contacts [58]) are more prone to do so and that such contacts also tend to be more repetitive and clustered, it is likely that random mixing models also overestimate the outbreak size for given *n*, *β *and *τ*. However, problems will definitely arise when the impact of social distancing measures (decrease of *n*) or of antiviral treatment (decrease of *β*) are estimated under the random mixing assumption: Both interventions will be much more effective in a more elaborate model than in a random mixing model when *n*, *β *and *τ *are the same for both model types. This argumentation is consistent with recent findings on the impact of other network properties on influenza spread: Heterogeneity in degree distribution does not influence the outbreak size in case of highly contagious influenza strains, but does so for moderately contagious strains; however, it does influence the total outbreak size when interventions are simulated – even in case of highly contagious strains [4].

## Conclusion

Real-world contact patterns are complex. They typically show all kinds of intermediate states ranging from contacts repeating on a daily basis to and never again. There are various clearly defined, cohesive groups with typically high intra-group clustering coefficients (e.g. households, workgroups, peer groups at school) and, at the same time, random contacts, e.g., in a leisure setting. Moreover, contacts differ in intensity and duration, which further complicates the dynamics of disease spread in such settings. This paper simplifies these complex patterns to a manageable model and parameter space that can be investigated systematically. Our research applies to diseases transmitted via conversational or direct contact, for which a typical number of contacts per day can be defined. For such diseases, our findings can help modellers judge whether a specific transmission system consisting of a specific infectious agent and a specific human system at risk can be represented by a simple random mixing model or if more elaborate models are necessary.

Random mixing models result in acceptable estimates of the total outbreak size  even if the real world contacts are highly repetitive and clustered

• if the number of potentially infectious contacts per day is high and

• if the transmission probability for a single infectious-susceptible pair is high and

• particularly, if the infectious period is just one to three days.

If the number of contacts per day or the transmission probability is low, particular consideration should be given to the actual structure of potentially contagious contacts in designing the model.

## Competing interests

The authors declare that they have no competing interests.

## Authors' contributions

TS carried out the majority of the model design, implemented the model, computed the analyses and prepared the manuscript as the lead writer. LF participated in the model design, contributed to the epidemiological interpretation of the model results, reviewed the literature on model parameters for specific diseases and helped to draft the manuscript. RWS participated in the model design and helped to draft the manuscript. All authors read and approved the final manuscript.

## Supplementary Material

Additional file 1 **Algorithms**. Provides a description of the key algorithms used for this paper following the ISO 5807-1985 standard. Click here for file

Additional file 2 **Epidemic curves**. This document provides exemplary epidemic curves for selected parameter settings. Click here for file

Additional file 3 **Contour plots & tables**. Additional contour plots for the differences in peak size and the differences in the simulation time till the peak is reached are given. In addition, data tables of means and standard deviations are provided for many analyses presented in this paper. Click here for file

Additional file 4 **Reproduction numbers**. This document shows how equation 1 can be derived. Click here for file
