# Supplementary material for: Models of epidemics: when contact repetition and clustering should be included
Source: Theor Biol Med Model. 2009 Jun 29;6:11. doi: 10.1186/1742-4682-6-11 (PMC2709892; doi:10.1186/1742-4682-6-11)

Additional file 1 – Algorithms

Table of contents

Algorithms used for this paper 1
Description of symbols used in this document 2
Contact generation for the random case..3
Contact generation for the repetitive case...4
SIR model 7

Algorithms used for this paper

We describe in this document the central algorithms on which the simulations of this paper base. The algorithms are given as flowcharts. These flowcharts are designed following the international standard ISO 5807-1985[1]. The symbols used in this document are briefly described below. Detailed descriptions can be found in the ISO 5807-1985 documentation.

Where necessary, subparts of the algorithms are described in detail. Straightforward operations are not described in detail but as generic processes.

In this supplementary material we describe the following algorithms:

- Generation of contacts in the random mixing case
- Generation of contacts in the repetitive contacts case (with clustering)
- SIR model

Algorithms used for other kinds of data handling, data presentation or storage and analysis are not presented.

The core of our simulation software is structured into two main processes: (1) the generation of contacts and (2) the simulation of disease spread.
The contact generation algorithms generates for each individual in the population pre-defined sets of n contact partners for τ different days, whereas n is the number of contacts per day and τ is the infectious period. There exist two version of the contact generation algorithm: one for the random mixing case and one for the case of repetitive contacts allowing for clustering. The generated contacts are stored in a contact list for each individual.
The algorithm for the disease spread makes use of these pre-defined contact list by simulating stochastically how a disease with given parameters spreads through such a given structure.

[1] ISO 5807-1985: Information processing – Documentation symbols and conventions for data, program and system flowcharts, program network charts and system resources charts.

Description of symbols used in this document

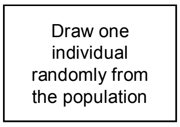

Process:
Used for any kind of defined operation or group of operations.

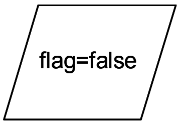

Data:
Used as representation of data

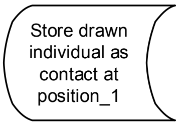

Stored data:
Used as representation of stored data

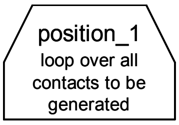

Begin of loop:
1st line indicates either a WHILE loop or a counter;
2nd line gives termination criterion

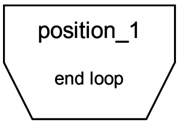

End of loop:
Go to begin of loop if termination criterion is not fulfilled

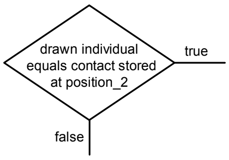

Decision:
Depending on whether the given statement is true or false, the one or theother path is used.

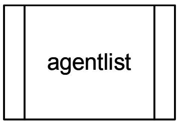

Predefined process:
Used for a defined process specified elsewhere in this document

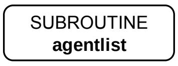

Terminator of a predefined process

Contact generation for the random case

Main routine for the generation of non-repetitive contacts:

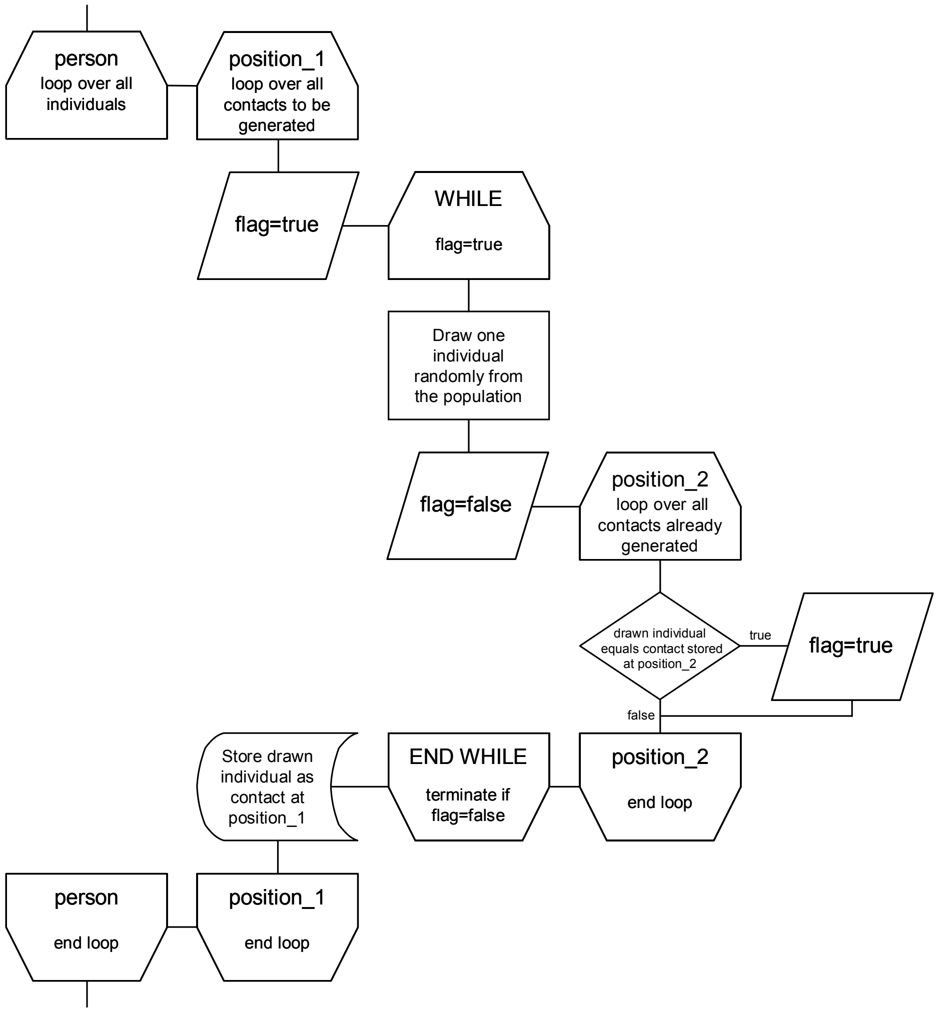

Contact generation for the repetitive case (allowing for clustering)

Main routine for the generation of repetitive contacts:

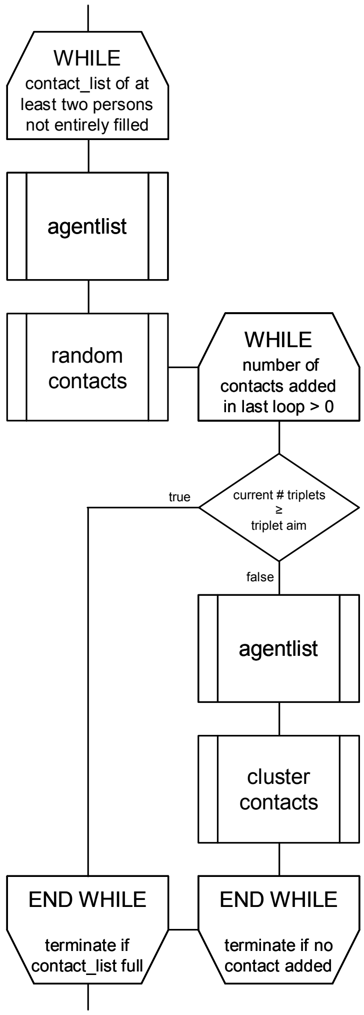

Subroutine “agentlist”:

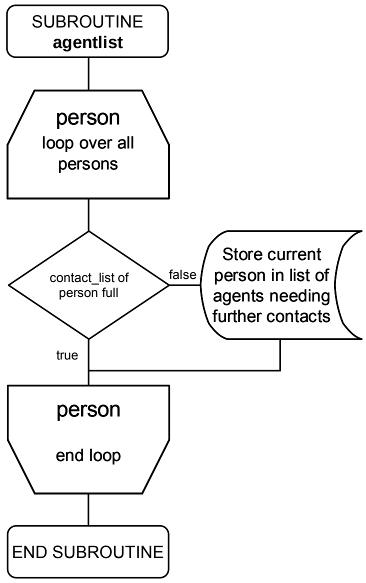

Subroutine „random contacts“:

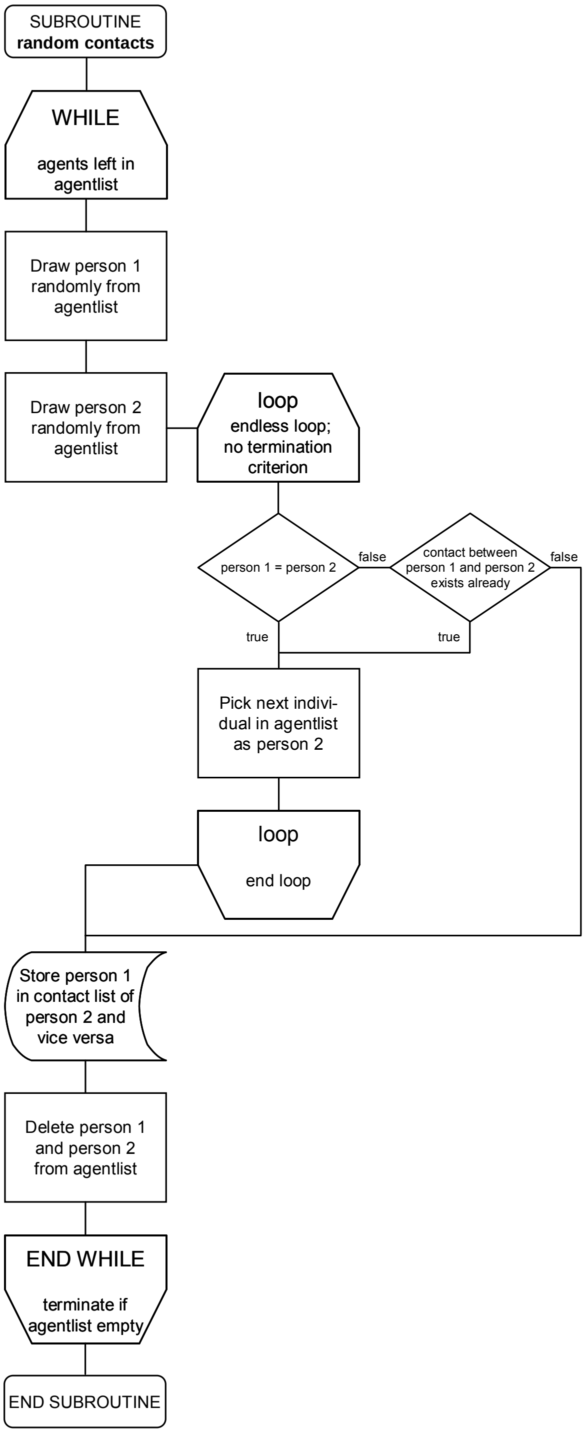

Subroutine „cluster contacts“:

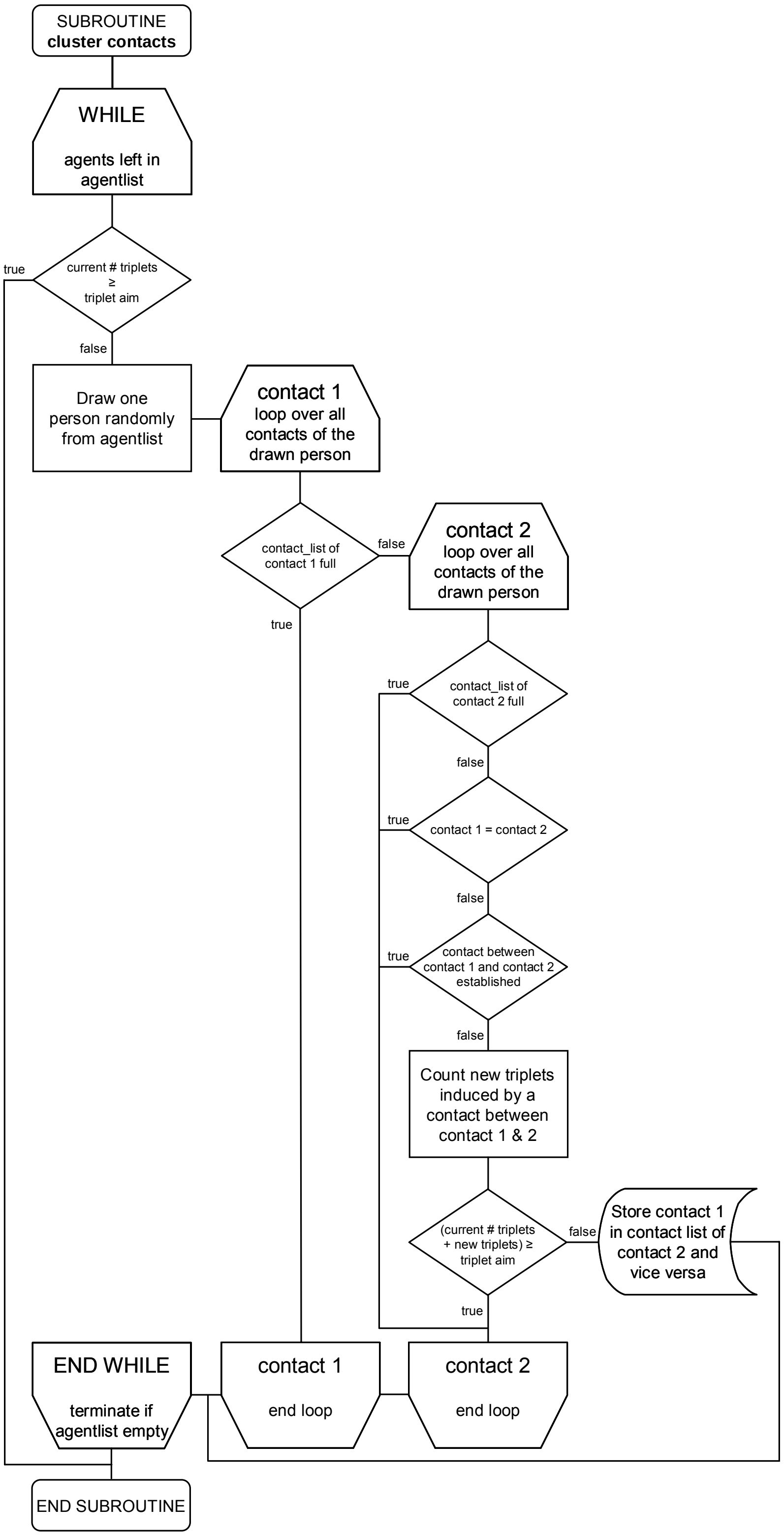

SIR model

Main routine for the simulation of disease spread:

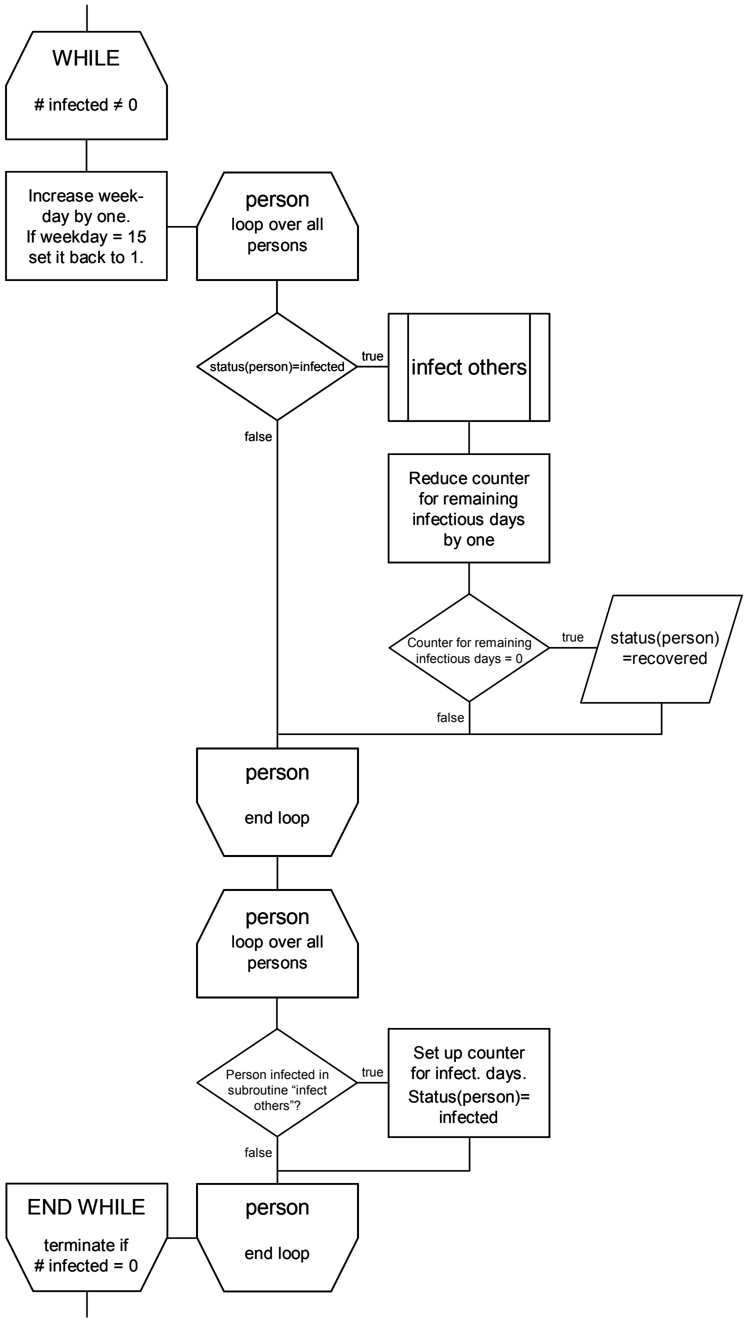

Subroutine „infect others“:

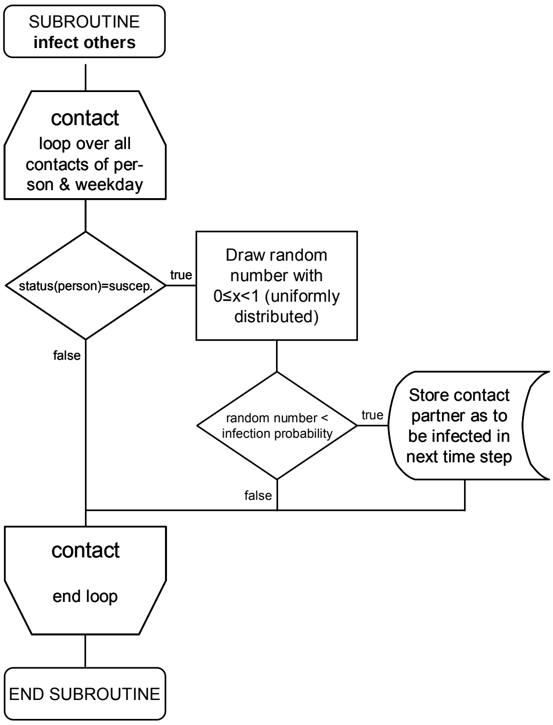

Supplement: Additional file 1 — Algorithms. Provides a description of the key algorithms used for this paper following the ISO 5807-1985 standard. [file 1742-4682-6-11-S1.pdf]
