# Supplementary material for: Models of epidemics: when contact repetition and clustering should be included
Source: Theor Biol Med Model. 2009 Jun 29;6:11. doi: 10.1186/1742-4682-6-11 (PMC2709892; doi:10.1186/1742-4682-6-11)
Supplement: Additional file 2 — Epidemic curves. This document provides exemplary epidemic curves for selected parameter settings. [file 1742-4682-6-11-S2.pdf]

Additional file 2 – Epidemic curves

In this document, we provide selected epidemic curves of simulation runs presented and discussed in our paper. The aim of this supplementary material is to give the reader an impression on dynamical differences between settings, which are compared regarding the aggregated indicator total outbreak size in the paper.

Table of contents

Legend..1
Curves depending on τ and $\beta \cdot n \cdot \tau$ (fixed $n = 4$ and no clustering) 1
Curves depending on n ($\tau = 8$ and $\beta \cdot n \cdot \tau = 1.6$ and no clustering)........................5
Epidemic curves and clustering 7

Legend

τ Infectious period [days]
n Number of contacts per day
β Probability of disease transmission per day for one infectious-susceptible pair
CC Clustering coefficient (for exact definition confer to the paper)

Curves depending on τ and $\beta \cdot n \cdot \tau$ (fixed $n = 4$ and no clustering)

In each of the figures 1-9 we present epidemic curves for 20 simulation runs for singular contacts and 20 simulation runs for repeated contacts under varied parameter conditions. Thereby, we show the 20 first runs of each parameter combination.

The qualitative impression of figures 1-9 is in total agreement with the quantitative, indicator-based findings of the main text and supplementary material 3: The larger the infectivity, the closer are the random mixing and the repetitive case. Differences in the infectious period do not lead to a visible qualitative change of the difference. The relative position of the peaks of both curves and the relative duration seem to be constant.

The following table gives an overview of the system behind figures 1-9

	$\beta \cdot n \cdot \tau = 4.0$	$\beta \cdot n \cdot \tau = 3.0$	$\beta \cdot n \cdot \tau = 1.6$
$\tau = 3$	Figure 1	Figure 4	Figure 7
$\tau = 8$	Figure 2	Figure 5	Figure 8
$\tau = 14$	Figure 3	Figure 6	Figure 9

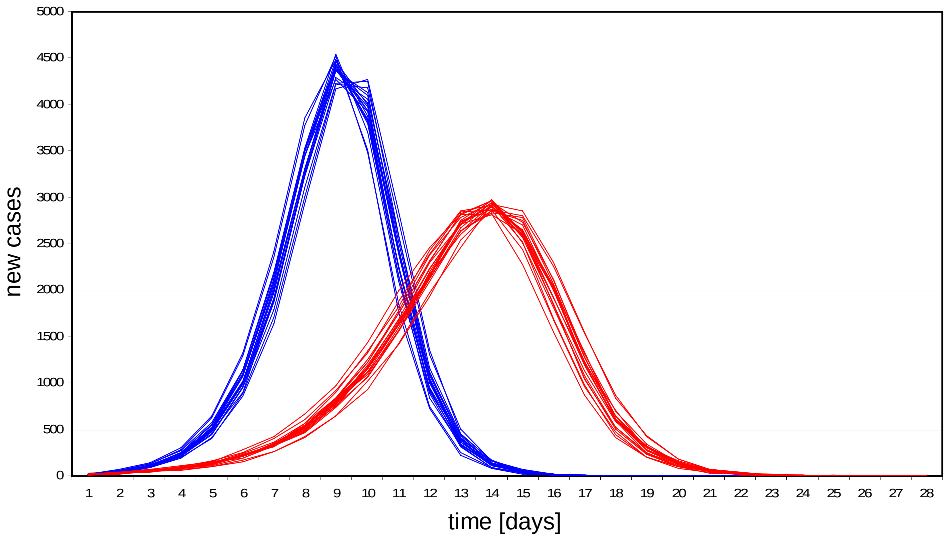

Figure 1 $\tau = 3$; $\beta \cdot n \cdot \tau = 4.0$; $n = 4$; **blue = singular cont.; red = repeated cont.**

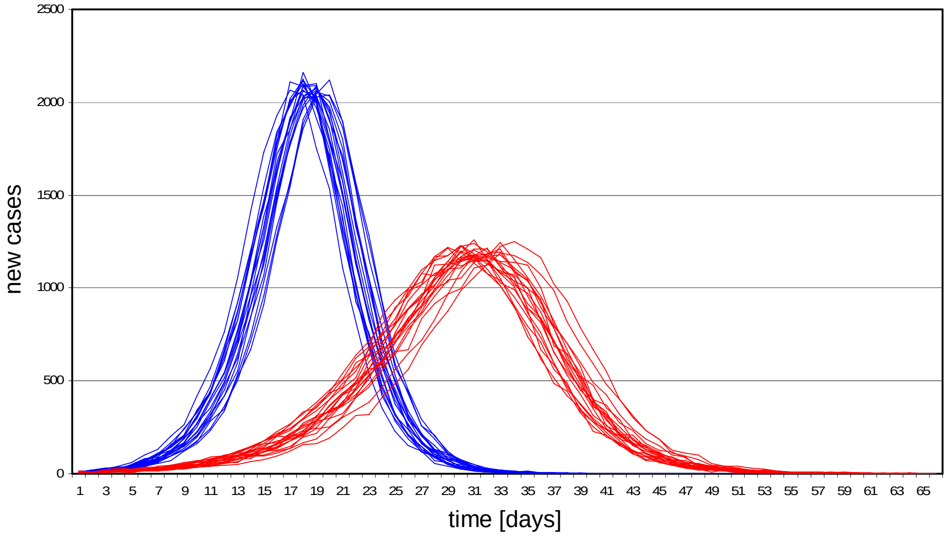

Figure 2 $\tau = 8$; $\beta \cdot n \cdot \tau = 4.0$; $n = 4$; **blue = singular cont.; red = repeated cont.**

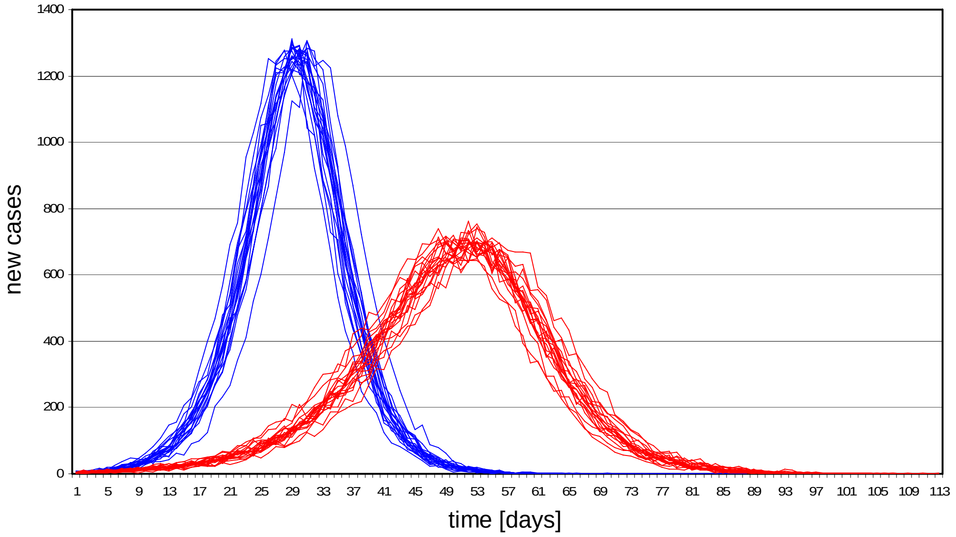

Figure 3 $\tau = 14$; $\beta \cdot n \cdot \tau = 4.0$; $n = 4$; **blue = singular cont.; red = repeated cont.**

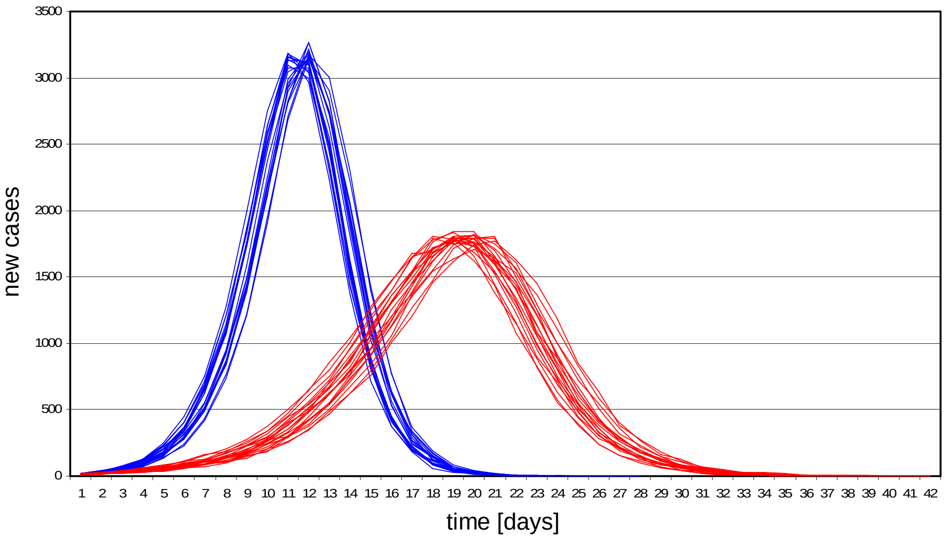

Figure 4 $\tau = 3$; $\beta \cdot n \cdot \tau = 3.0$; $n = 4$; **blue = singular cont.; red = repeated cont.**

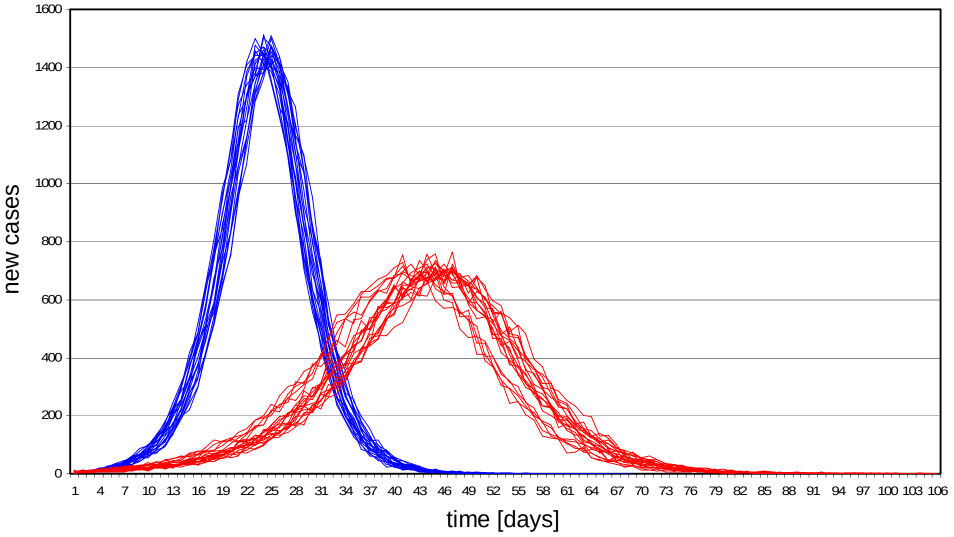

Figure 5 $\tau = 8$; $\beta \cdot n \cdot \tau = 3.0$; $n = 4$; **blue = singular cont.; red = repeated cont.**

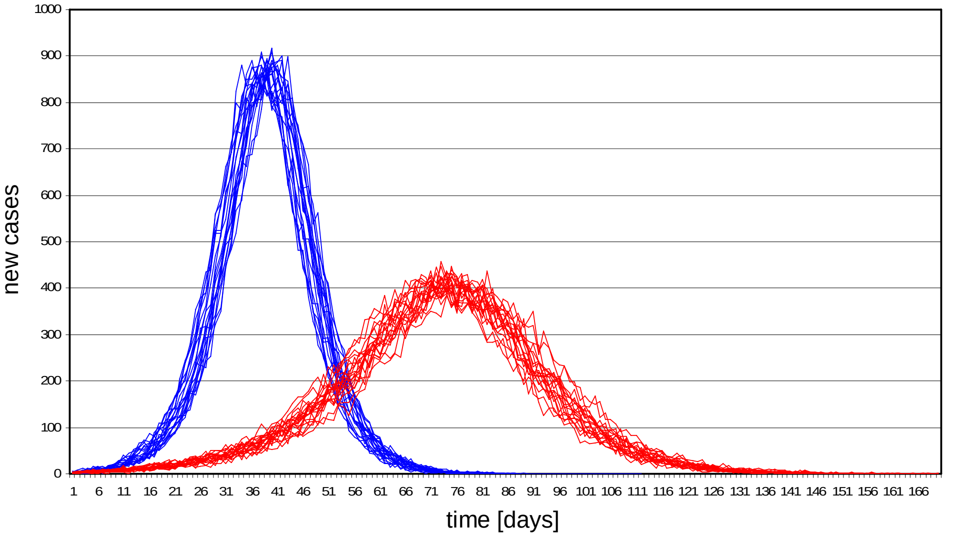

Figure 6 $\tau = 14$; $\beta \cdot n \cdot \tau = 3.0$; $n = 4$; **blue = singular cont.; red = repeated cont.**

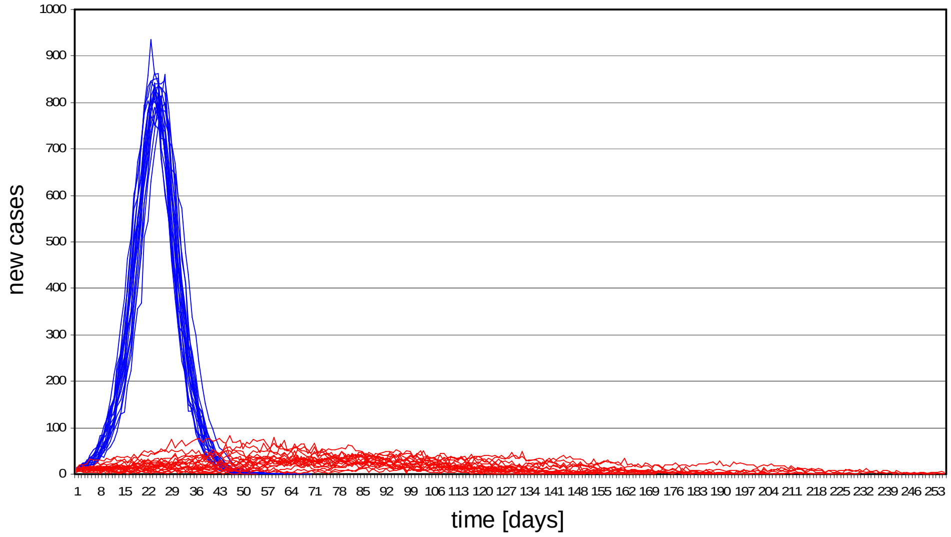

Figure 7 $\tau = 3$; $\beta \cdot n \cdot \tau = 1.6$; $n = 4$; **blue = singular cont.; red = repeated cont.**

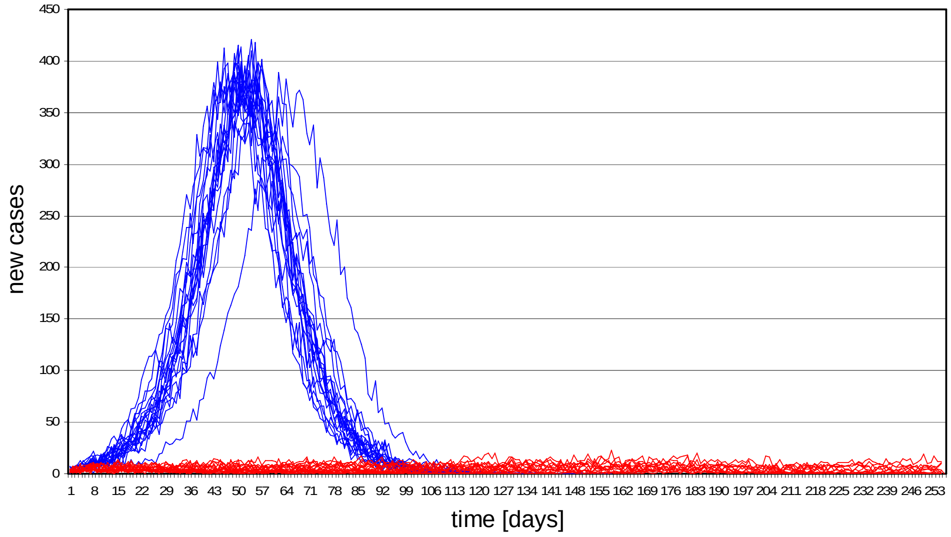

Figure 8 $\tau = 8$; $\beta \cdot n \cdot \tau = 1.6$; $n = 4$; **blue = singular cont.; red = repeated cont.**

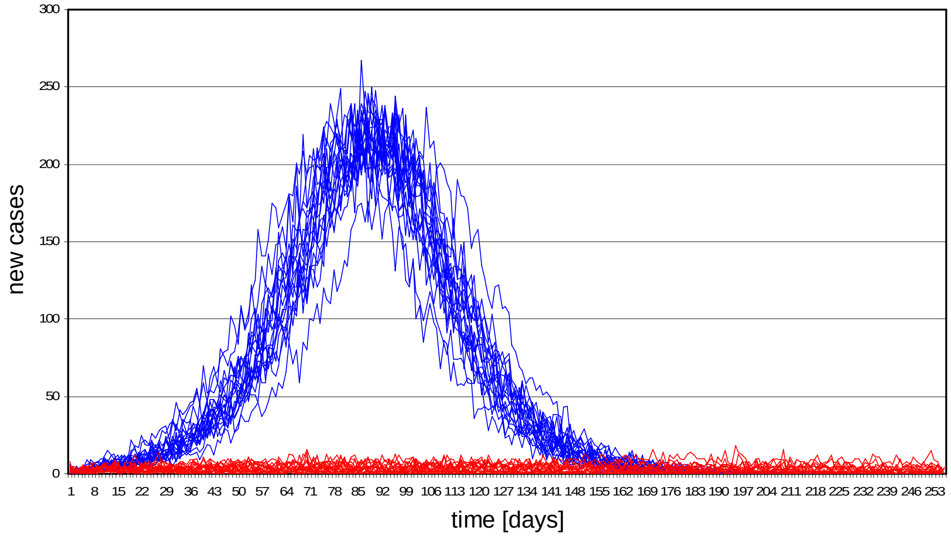

Figure 9 $\tau = 14$; $\beta \cdot n \cdot \tau = 1.6$; $n = 4$; **blue = singular cont.; red = repeated cont.**

Curves depending on n ($\tau = 8$ and $\beta \cdot n \cdot \tau = 1.6$ and no clustering)

In each of the figures 10-14 we present epidemic curves for 20 simulation runs for singular contacts and 20 simulation runs for repeated contacts for fixed τ and $\beta \cdot n \cdot \tau$, but increasing n. The fixed parameters have been chosen in a way that maximal changes for varying n can be expected.

Figures 10-14 show expectedly, that the differences between runs with singular contacts and runs with repetitive contacts are larges for small n. For large numbers of contacts per day, the model with repeating contacts behaves similar to a model with daily changing contacts.

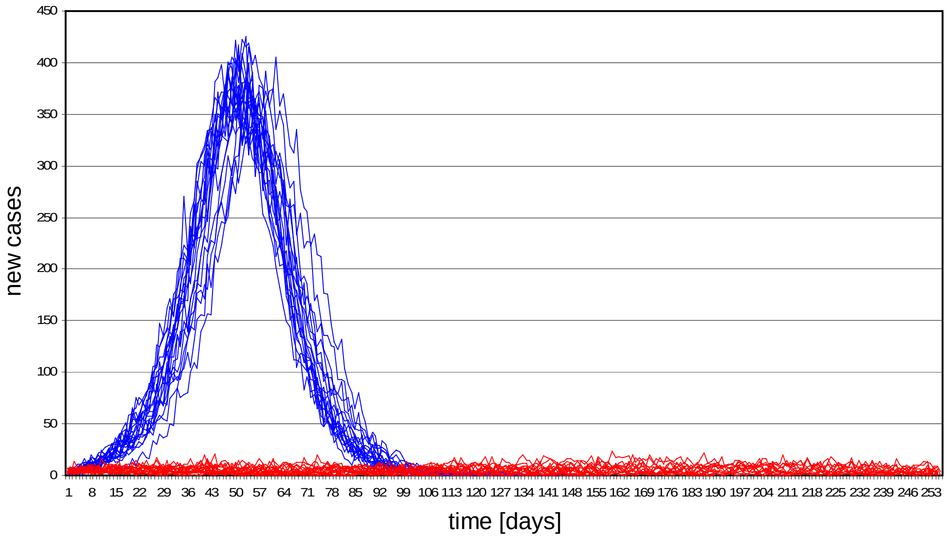

Figure 10 $n = 4$; $\tau = 8$; $\beta \cdot n \cdot \tau = 1.6$; **blue = singular cont.; red = repeated cont.**

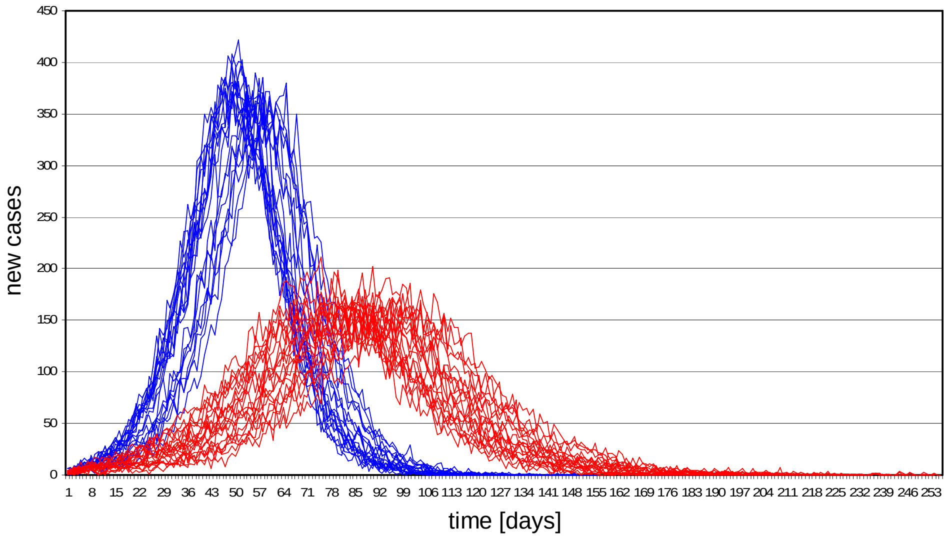

Figure 11 $n = 8$; $\tau = 8$; $\beta \cdot n \cdot \tau = 1.6$; **blue = singular cont.; red = repeated cont.**

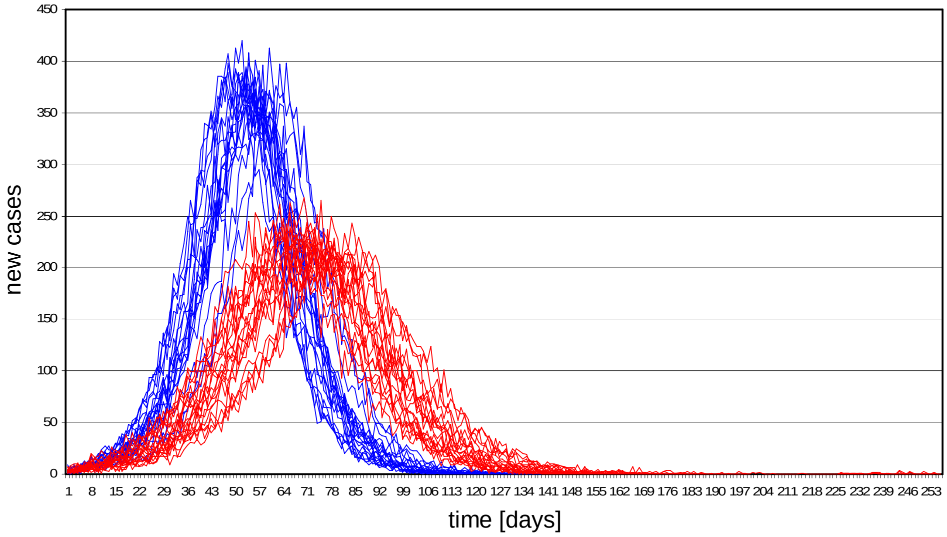

Figure 12 $n = 12$; $\tau = 8$; $\beta \cdot n \cdot \tau = 1.6$; **blue = singular cont.; red = repeated cont.**

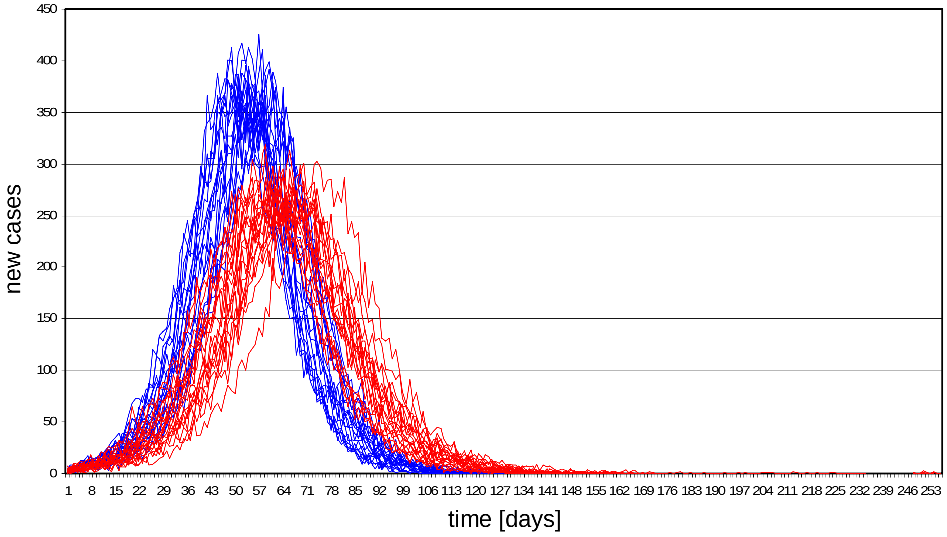

Figure 13 $n = 16$; $\tau = 8$; $\beta \cdot n \cdot \tau = 1.6$; **blue = singular cont.; red = repeated cont.**

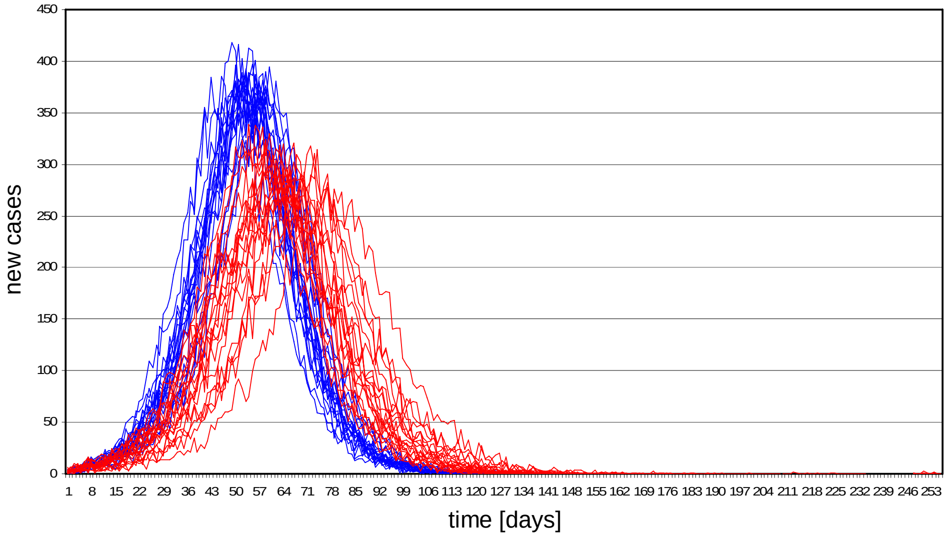

Figure 14 $n = 20$; $\tau = 8$; $\beta \cdot n \cdot \tau = 1.6$; **blue = singular cont.; red = repeated cont.**

Epidemic curves and clustering

Figure 15 shows the effect of clustering on epidemic curves by otherwise identical parameter conditions. All curves have been calculated for the case of daily repeating contacts. The blue lines show the epidemic curves of the first 20 runs of a model with $n = 12$; $\tau = 14$; $\beta \cdot n \cdot \tau = 1.8$ and no clustering. The red lines show the curves for a model with identical parameter conditions, but a clustering coefficient of 20%. The green lines are based on runs with a clustering coefficient of 40%. The bold lines are averaged values while the thin lines represent single simulation runs.

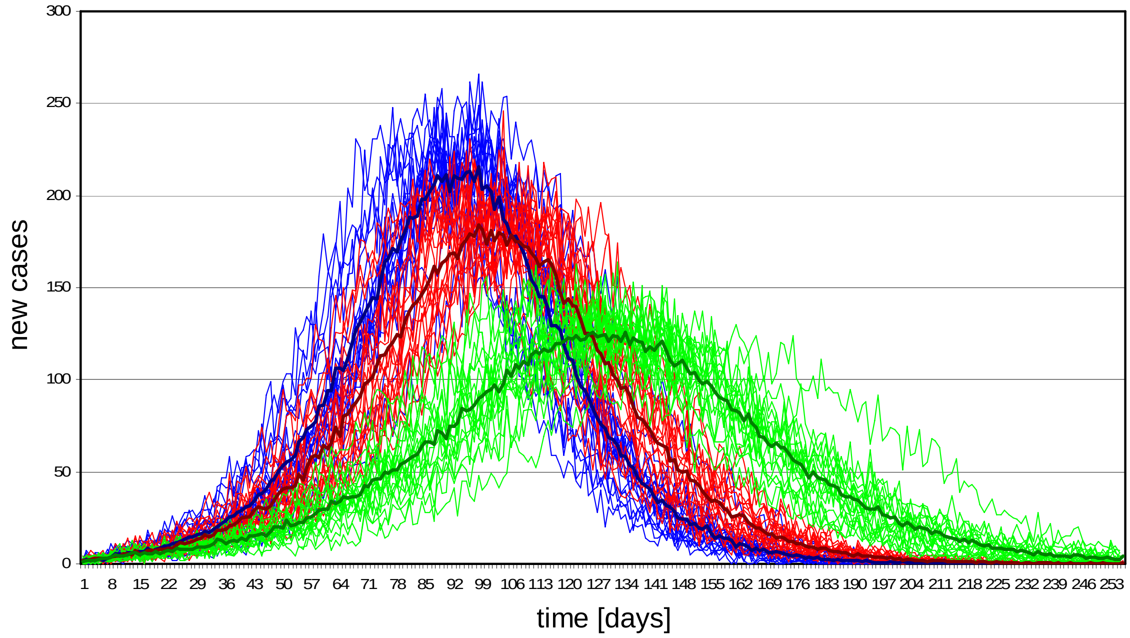

Figure 15 $n = 12$ **;** $\tau = 14$ **;** $\beta \cdot n \cdot \tau = 1.8$ **; blue:** $CC = 0.0$ **; red:** 0.2 **; green:** 0.4 **.**
