# Supplementary material for: Models of epidemics: when contact repetition and clustering should be included
Source: Theor Biol Med Model. 2009 Jun 29;6:11. doi: 10.1186/1742-4682-6-11 (PMC2709892; doi:10.1186/1742-4682-6-11)
Supplement: Additional file 3 — Contour plots & tables. Additional contour plots for the differences in peak size and the differences in the simulation time till the peak is reached are given. In addition, data tables of means and standard deviations are provided for many analyses presented in this paper. [file 1742-4682-6-11-S3.pdf]

Additional file 3 – Contour plots & tables

Table of contents

Legend..1
Contour plots for $I_{\max}$ 2
Contour plots for $t_{I\max}$ 4
Data tables for I_{tot} 6
Data tables for $I_{\max}$ 13
Data tables for $t_{I\max}$ 18

Legend

τ	Infectious period [days]
n	Number of contacts per day
β	Probability of disease transmission per day for one infectious-susceptible pair
CC	Clustering coefficient (for definition see paper)
$_{ran}$	Index denoting the random mixing case: Contact partners are randomly chosen from the entire population, change every day and do not repeat
$_{rep}$	Index standing for repetitive contacts, i.e. that the contact partners of every individual repeat every day during the entire simulation run.
I_{tot}	Total outbreak size (= total number of individuals infected during a run)
$I_{\max}$	Value at peak of epidemic curve (equals $\Delta S/\Delta t$)
$t_{I\max}$	Time from $t=0$ to $I_{\max}$
M	Mean value
SD	Standard deviation

Contour plots for $I_{\max}$

The following contour plots show the difference between $\bar{I}_{\max,ran}$ and $\bar{I}_{\max,rep}$ relative to $\bar{I}_{\max,ran}$. Hence, the scale is different from the scale used in Figures 2 and 4 of the main paper!

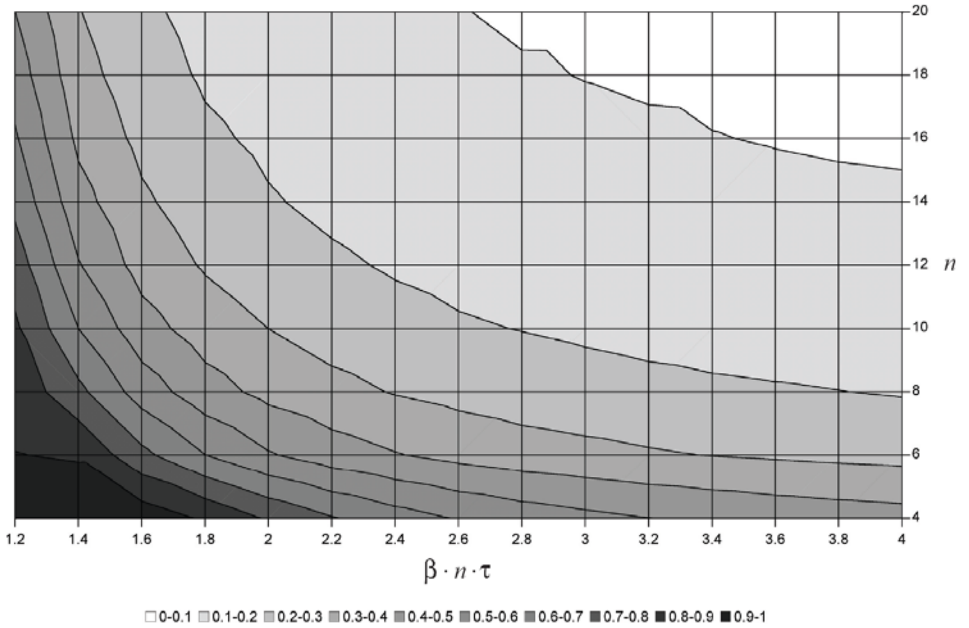

Figure 1 $\left(\bar{I}_{\max,ran} - \bar{I}_{\max,rep}\right) / \bar{I}_{\max,ran}$ **with** $\tau = 14$ **and** $CC = .0$

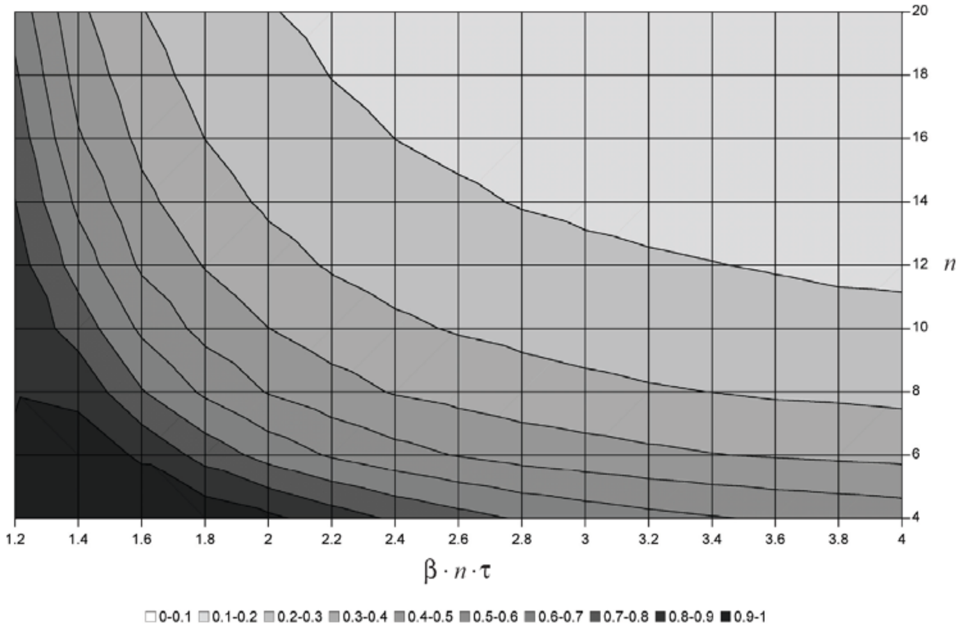

Figure 2 $\left(\bar{I}_{\max,ran} - \bar{I}_{\max,rep}\right) / \bar{I}_{\max,ran}$ **with** $\tau = 14$ **and** $CC = .2$

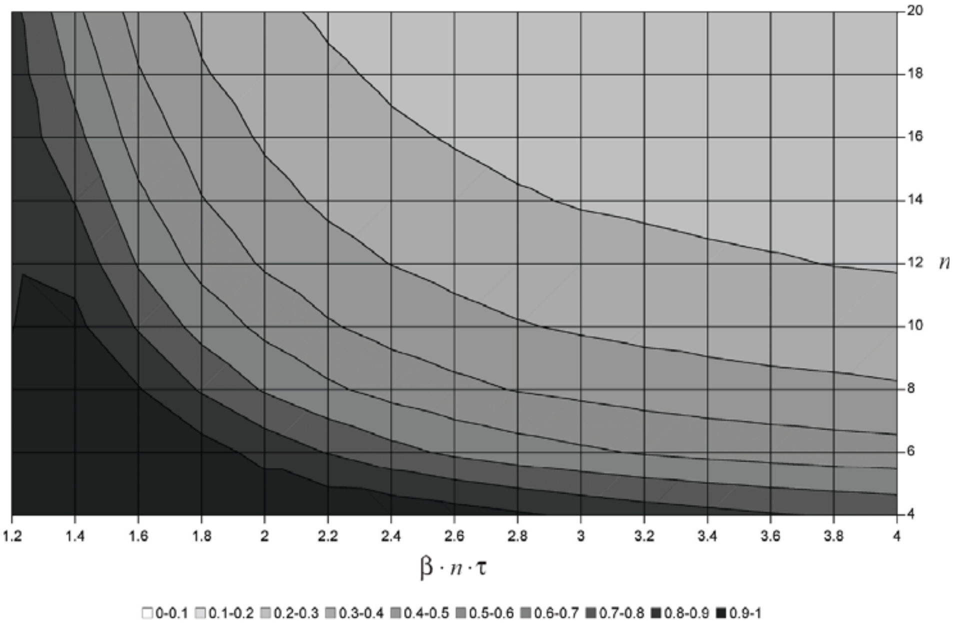

Figure 3 $\left(\bar{I}_{\max,ran} - \bar{I}_{\max,rep}\right) / \bar{I}_{\max,ran}$ **with** $\tau = 14$ **and** $CC = .4$

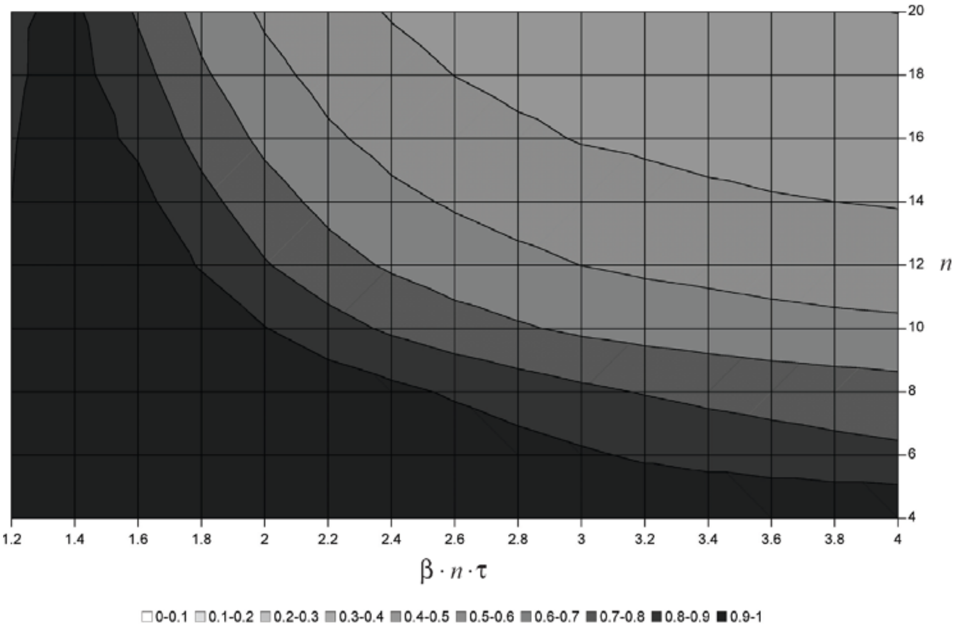

Figure 4 $\left(\bar{I}_{\max,ran} - \bar{I}_{\max,rep}\right) / \bar{I}_{\max,ran}$ **with** $\tau = 14$ **and** $CC = .6$

Contour plots for $t_{I\max}$

The following contour plots show the difference between $\bar{t}_{I\max,\text{rep}}$ and $\bar{t}_{I\max,\text{ran}}$ relative to $\bar{t}_{I\max,\text{rep}}$. Hence, the scale is different from the scale used in Figures 2 and 4 of the main paper! Areas, in which $\bar{t}_{I\max,\text{rep}}$ is smaller than $\bar{t}_{I\max,\text{ran}}$, are covered with a striped area, because for these cases in many simulation runs no clear outbreak with a clear peak could be observed. Without a typically shaped epidemic curve, the indicitor $\bar{t}_{I\max}$ is not well defined and arbitrary in its value. The corresponding data are given in the tables.

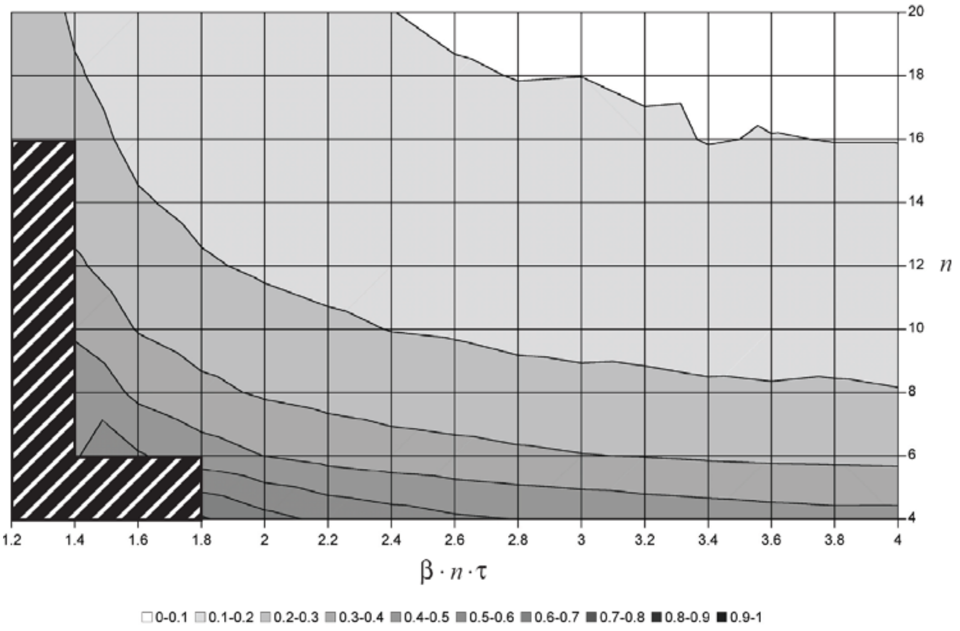

Figure 5 $\left(\bar{t}_{I\max,\text{rep}} - \bar{t}_{I\max,\text{ran}}\right)/\bar{t}_{I\max,\text{rep}}$ **with** $\tau = 14$ **and** $CC = .0$

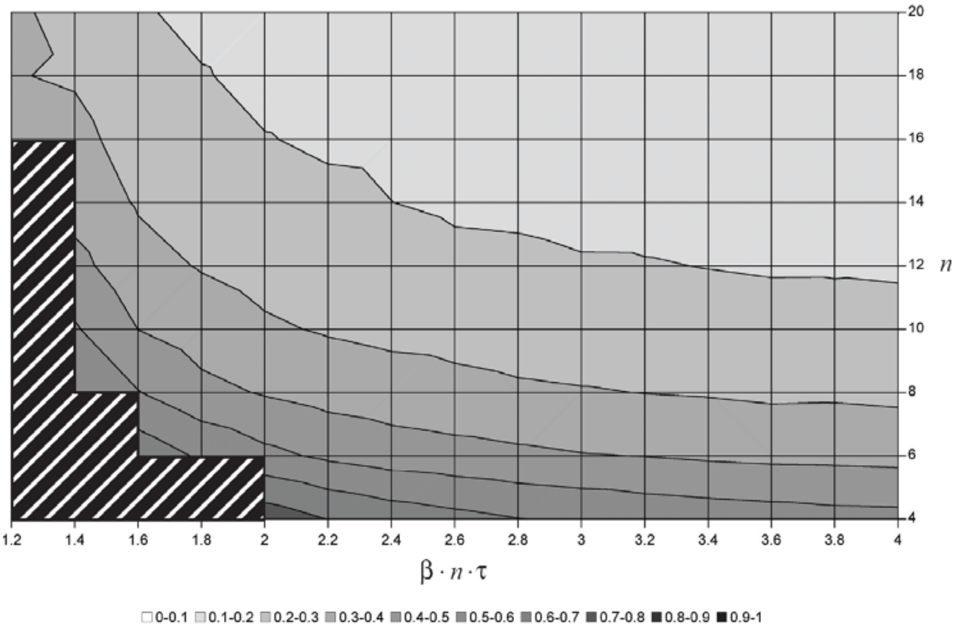

Figure 6 $\left(\bar{t}_{I\max,\text{rep}} - \bar{t}_{I\max,\text{ran}}\right)/\bar{t}_{I\max,\text{rep}}$ **with** $\tau = 14$ **and** $CC = .2$

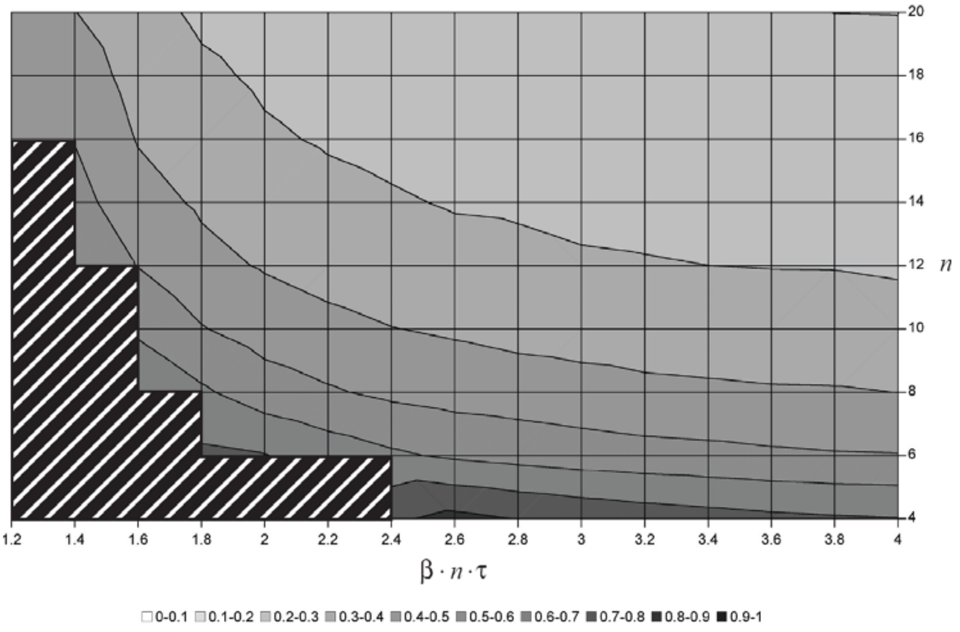

Figure 7 $\left(\bar{t}_{I\max,\mathrm{rep}} - \bar{t}_{I\max,\mathrm{ran}}\right)/\bar{t}_{I\max,\mathrm{rep}}$ **with** $\tau = 14$ **and** $CC = .4$

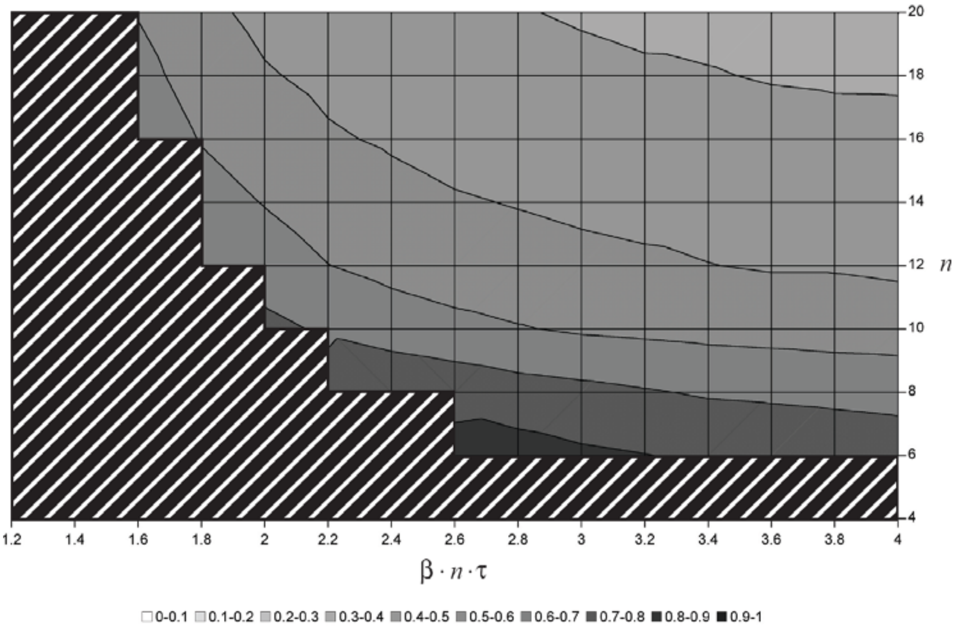

Figure 8 $\left(\bar{t}_{I\max,\mathrm{rep}} - \bar{t}_{I\max,\mathrm{ran}}\right)/\bar{t}_{I\max,\mathrm{rep}}$ **with** $\tau = 14$ **and** $CC = .6$

Data tables for I_{tot}

Mean values and standard deviation of $I_{tot,ran}$ for $4 \le n \le 20$; $1.2 \le \beta \cdot n \cdot \tau \le 4.0$; $\tau = 14$

n		$\beta \cdot n \cdot \tau$														
		**1.2**	**1.4**	**1.6**	**1.8**	**2.0**	**2.2**	**2.4**	**2.6**	**2.8**	**3.0**	**3.2**	**3.4**	**3.6**	**3.8**	**4.0**
**4**	M	6266.2	10230.6	12827.3	14640.6	15930.0	16861.4	17555.5	18086.6	18484.0	18794.2	19036.2	19226.0	19374.7	19494.3	19589.4
	SD	*484.5*	*224.4*	*160.8*	*125.7*	*95.4*	*76.7*	*64.3*	*54.7*	*48.4*	*40.9*	*32.5*	*29.3*	*27.9*	*23.9*	*21.4*
**6**	M	6219.0	10213.8	12838.8	14642.7	15925.4	16862.0	17560.1	18086.6	18484.5	18794.7	19034.8	19227.3	19376.5	19493.6	19588.2
	SD	*684.0*	*211.0*	*153.4*	*121.2*	*88.4*	*83.0*	*66.8*	*54.6*	*46.2*	*39.6*	*35.1*	*31.1*	*26.5*	*23.7*	*22.1*
**8**	M	6218.0	10222.5	12845.6	14633.2	15925.5	16871.0	17555.4	18085.8	18489.4	18798.2	19036.8	19225.5	19375.0	19493.8	19588.7
	SD	*681.8*	*227.4*	*154.4*	*120.0*	*95.9*	*80.2*	*66.7*	*54.2*	*49.1*	*41.4*	*37.0*	*31.7*	*29.0*	*24.4*	*21.0*
**10**	M	6252.1	10211.3	12844.6	14635.2	15930.4	16861.2	17557.9	18086.1	18483.4	18792.4	19038.0	19225.0	19375.7	19495.1	19588.6
	SD	*596.7*	*228.0*	*161.8*	*120.8*	*91.3*	*74.1*	*63.3*	*56.4*	*47.4*	*41.7*	*36.7*	*31.4*	*26.4*	*24.3*	*21.4*
**12**	M	6272.1	10223.9	12826.9	14644.8	15919.7	16863.0	17561.8	18083.7	18484.7	18796.2	19037.1	19227.2	19377.3	19496.0	19589.5
	SD	*498.3*	*223.9*	*159.1*	*120.9*	*97.9*	*78.0*	*62.5*	*54.2*	*50.0*	*41.5*	*35.4*	*33.1*	*26.2*	*23.6*	*20.5*
**14**	M	6285.0	10223.7	12824.8	14645.0	15922.3	16863.2	17556.4	18086.4	18483.6	18796.8	19036.9	19226.2	19376.5	19494.0	19592.0
	SD	*503.9*	*233.6*	*175.5*	*121.1*	*97.0*	*78.0*	*68.2*	*54.1*	*46.7*	*39.3*	*34.6*	*31.7*	*26.4*	*24.1*	*23.3*
**16**	M	6246.3	10220.2	12834.0	14639.8	15932.1	16859.4	17559.7	18083.0	18487.2	18795.5	19038.4	19224.7	19377.2	19494.0	19588.0
	SD	*602.7*	*223.2*	*173.6*	*120.8*	*99.6*	*79.4*	*67.8*	*54.3*	*48.1*	*41.1*	*35.5*	*28.3*	*26.9*	*23.8*	*21.3*
**18**	M	6216.6	10225.4	12841.6	14636.0	15923.7	16865.7	17556.2	18085.4	18488.8	18793.7	19038.6	19223.8	19374.9	19492.9	19590.0
	SD	*763.7*	*231.3*	*161.4*	*121.8*	*99.6*	*80.9*	*66.1*	*54.8*	*47.2*	*41.1*	*34.8*	*33.8*	*26.0*	*24.1*	*22.3*
**20**	M	6202.1	10216.7	12831.5	14641.5	15921.3	16862.8	17558.6	18087.1	18484.5	18798.1	19035.6	19229.2	19375.4	19495.5	19589.1
	SD	*831.3*	*239.2*	*171.4*	*122.2*	*96.9*	*76.0*	*64.6*	*58.8*	*51.8*	*42.6*	*35.8*	*29.1*	*28.1*	*25.3*	*19.9*

Mean values and standard deviation of $I_{tot,rep}$ for $4 \le n \le 20$; $1.2 \le \beta \cdot n \cdot \tau \le 4.0$; $\tau = 14$; $CC = 0.0$

n		$\beta \cdot n \cdot \tau$														
		**1.2**	**1.4**	**1.6**	**1.8**	**2.0**	**2.2**	**2.4**	**2.6**	**2.8**	**3.0**	**3.2**	**3.4**	**3.6**	**3.8**	**4.0**
**4**	M	73.0	167.2	958.5	6581.8	10773.3	13449.8	15278.8	16576.8	17459.6	18102.7	18574.7	18913.4	19167.5	19361.8	19503.9
	SD	*34.9*	*98.0*	*718.8*	*682.0*	*337.1*	*230.4*	*155.4*	*122.5*	*92.9*	*78.2*	*60.0*	*49.7*	*40.7*	*35.7*	*29.8*
**6**	M	170.6	2423.9	8140.8	11598.0	13950.1	15547.7	16678.7	17499.9	18091.8	18536.0	18865.9	19115.7	19307.1	19453.7	19566.8
	SD	*120.7*	*1166.6*	*392.8*	*245.1*	*170.1*	*126.6*	*99.0*	*75.9*	*67.0*	*54.0*	*46.0*	*35.8*	*32.2*	*28.0*	*24.4*
**8**	M	456.3	5689.7	10000.0	12819.0	14718.2	16064.2	17027.0	17731.8	18250.8	18641.3	18937.2	19166.0	19340.9	19474.8	19581.4
	SD	*401.4*	*603.4*	*276.5*	*191.7*	*147.9*	*103.6*	*90.2*	*67.0*	*60.1*	*49.3*	*38.4*	*34.9*	*32.4*	*28.5*	*24.6*
**10**	M	1088.7	7003.8	10815.0	13344.5	15069.0	16292.8	17184.3	17834.1	18321.3	18686.6	18968.5	19182.4	19353.1	19482.2	19585.6
	SD	*844.4*	*539.0*	*258.1*	*171.5*	*120.1*	*102.1*	*81.5*	*63.2*	*53.7*	*49.0*	*41.3*	*33.2*	*29.1*	*25.7*	*21.6*
**12**	M	1920.2	7748.6	11284.3	13620.2	15266.3	16426.5	17260.8	17891.3	18358.7	18713.7	18986.0	19197.8	19358.9	19486.6	19586.1
	SD	*1098.8*	*366.7*	*221.2*	*161.0*	*123.4*	*94.4*	*75.8*	*61.7*	*52.2*	*46.8*	*40.2*	*32.6*	*29.3*	*24.4*	*23.1*
**14**	M	2692.1	8224.9	11562.5	13806.4	15388.5	16498.4	17323.6	17924.0	18384.2	18732.0	18994.9	19199.7	19360.5	19491.6	19587.1
	SD	*1116.2*	*339.4*	*200.2*	*158.6*	*117.1*	*91.2*	*72.8*	*64.5*	*50.6*	*45.0*	*36.8*	*33.3*	*27.9*	*25.7*	*22.2*
**16**	M	3288.4	8533.0	11756.7	13948.3	15465.7	16562.4	17356.0	17955.8	18396.9	18741.2	18998.7	19207.2	19365.2	19488.3	19587.9
	SD	*1075.8*	*304.0*	*208.3*	*153.2*	*115.3*	*90.4*	*77.9*	*62.3*	*50.6*	*43.1*	*35.7*	*32.1*	*28.2*	*25.9*	*21.6*
**18**	M	3698.2	8777.8	11902.1	14041.3	15532.6	16600.4	17386.2	17967.1	18411.5	18745.1	19006.8	19210.1	19366.8	19490.2	19588.9
	SD	*1035.9*	*281.7*	*200.2*	*148.9*	*115.6*	*87.5*	*70.3*	*63.6*	*49.4*	*42.7*	*38.9*	*32.8*	*26.9*	*25.8*	*22.3*
**20**	M	4072.7	8926.7	12017.0	14103.7	15580.8	16634.4	17403.6	17985.3	18424.1	18751.1	19012.8	19209.9	19368.5	19491.5	19589.4
	SD	*946.3*	*292.8*	*196.2*	*147.4*	*115.6*	*88.1*	*74.5*	*62.2*	*49.5*	*45.6*	*36.8*	*31.9*	*28.9*	*24.8*	*20.8*

Mean values and standard deviation of $I_{tot,rep}$ for $4 \le n \le 20$; $1.2 \le \beta \cdot n \cdot \tau \le 4.0$; $\tau = 14$; $CC = 0.2$

n		$\beta \cdot n \cdot \tau$														
		**1.2**	**1.4**	**1.6**	**1.8**	**2.0**	**2.2**	**2.4**	**2.6**	**2.8**	**3.0**	**3.2**	**3.4**	**3.6**	**3.8**	**4.0**
**4**	M	59.8	97.8	210.4	1154.2	6396.9	10530.4	13253.8	15131.6	16474.4	17414.2	18099.3	18571.0	18931.4	19189.8	19388.8
	SD	*26.6*	*39.4*	*124.6*	*841.6*	*851.0*	*400.9*	*279.4*	*198.6*	*162.5*	*112.4*	*84.6*	*78.8*	*60.6*	*49.8*	*39.0*
**6**	M	105.4	418.2	4905.8	9804.7	12814.6	14881.7	16265.6	17230.4	17924.5	18423.1	18797.9	19074.0	19277.6	19433.0	19554.3
	SD	*53.0*	*326.7*	*951.1*	*368.8*	*247.2*	*170.8*	*124.3*	*95.1*	*78.0*	*60.3*	*53.4*	*41.6*	*34.3*	*31.0*	*26.1*
**8**	M	196.8	2914.0	8538.1	11949.6	14212.2	15744.6	16838.1	17607.3	18182.3	18594.1	18911.4	19146.0	19328.8	19464.9	19574.1
	SD	*141.4*	*1196.2*	*581.5*	*236.8*	*175.8*	*126.6*	*97.1*	*77.8*	*62.3*	*53.9*	*44.3*	*38.3*	*32.1*	*26.8*	*24.3*
**10**	M	398.8	5382.6	9920.1	12824.1	14759.4	16109.6	17072.3	17763.7	18282.4	18666.2	18953.1	19175.2	19345.9	19478.9	19581.9
	SD	*360.6*	*680.3*	*299.0*	*200.3*	*136.6*	*110.9*	*85.8*	*73.3*	*58.4*	*52.1*	*40.3*	*35.0*	*31.2*	*25.0*	*22.4*
**12**	M	759.0	6583.9	10631.6	13252.2	15047.6	16300.4	17189.1	17849.1	18328.2	18694.7	18977.2	19190.9	19354.5	19484.7	19583.8
	SD	*649.8*	*583.2*	*260.2*	*184.8*	*136.5*	*99.2*	*80.5*	*67.3*	*53.8*	*49.1*	*41.6*	*33.9*	*30.4*	*27.0*	*21.7*
**14**	M	1269.4	7317.8	11070.4	13527.0	15225.9	16409.3	17268.0	17891.4	18366.7	18715.9	18987.8	19194.7	19361.5	19487.1	19586.6
	SD	*884.9*	*382.9*	*244.9*	*172.6*	*121.3*	*97.1*	*79.4*	*60.5*	*51.3*	*45.2*	*37.7*	*33.5*	*29.1*	*25.9*	*22.6*
**16**	M	1804.9	7824.6	11360.1	13710.4	15349.1	16488.1	17314.0	17929.4	18383.6	18734.6	18997.1	19203.6	19362.5	19488.9	19588.3
	SD	*1081.3*	*342.4*	*234.3*	*165.1*	*116.9*	*93.2*	*76.4*	*63.4*	*53.9*	*49.4*	*39.4*	*33.2*	*29.0*	*24.9*	*21.6*
**18**	M	2332.5	8137.2	11570.2	13853.9	15424.2	16539.7	17353.1	17944.8	18397.1	18738.8	19005.8	19205.1	19365.2	19488.7	19587.7
	SD	*1172.2*	*542.7*	*220.7*	*154.8*	*128.5*	*94.0*	*73.4*	*60.3*	*50.4*	*46.8*	*39.6*	*33.6*	*28.1*	*24.8*	*21.2*
**20**	M	2920.7	8422.4	11721.7	13949.9	15491.1	16582.2	17376.6	17966.9	18410.6	18745.5	19012.6	19207.5	19364.6	19489.9	19589.3
	SD	*1070.8*	*316.5*	*217.1*	*161.8*	*118.1*	*95.0*	*70.6*	*61.3*	*53.5*	*45.0*	*37.6*	*31.2*	*29.3*	*24.8*	*22.2*

Mean values and standard deviation of $I_{tot,rep}$ for $4 \le n \le 20$; $1.2 \le \beta \cdot n \cdot \tau \le 4.0$; $\tau = 14$; $CC = 0.4$

n		$\beta \cdot n \cdot \tau$														
		**1.2**	**1.4**	**1.6**	**1.8**	**2.0**	**2.2**	**2.4**	**2.6**	**2.8**	**3.0**	**3.2**	**3.4**	**3.6**	**3.8**	**4.0**
**4**	M	41.9	64.2	91.6	144.9	267.3	610.9	2883.5	7401.5	10671.3	13068.8	14819.0	16179.9	17142.4	17873.6	18413.9
	SD	*14.8*	*23.0*	*32.7*	*57.7*	*137.0*	*382.9*	*1259.6*	*717.3*	*490.2*	*346.6*	*271.7*	*222.2*	*175.6*	*138.6*	*128.4*
**6**	M	71.8	138.4	409.9	3573.0	8956.6	12348.0	14649.8	16164.5	17243.6	17974.6	18509.6	18869.6	19155.1	19354.5	19500.3
	SD	*32.8*	*69.0*	*269.6*	*1275.3*	*668.8*	*299.0*	*207.5*	*167.9*	*122.4*	*90.8*	*76.7*	*61.4*	*48.9*	*38.7*	*33.4*
**8**	M	106.7	371.3	4246.6	9482.8	12739.3	14884.6	16305.2	17283.3	17979.9	18475.6	18834.4	19099.0	19300.1	19448.9	19566.7
	SD	*52.8*	*269.9*	*1047.7*	*407.0*	*263.0*	*183.2*	*126.0*	*103.0*	*80.6*	*58.7*	*49.5*	*39.8*	*35.6*	*29.2*	*25.0*
**10**	M	155.5	1289.9	7466.3	11441.2	13982.8	15642.7	16798.8	17612.5	18191.7	18607.8	18925.7	19155.4	19331.5	19472.7	19579.5
	SD	*101.4*	*905.0*	*511.2*	*299.5*	*185.7*	*134.8*	*107.0*	*84.1*	*64.9*	*53.3*	*46.6*	*36.7*	*30.9*	*27.4*	*21.7*
**12**	M	224.2	3287.6	8953.6	12343.8	14513.4	16006.9	17025.5	17745.8	18278.2	18663.2	18954.3	19178.7	19348.3	19480.9	19585.1
	SD	*182.8*	*1167.4*	*401.2*	*242.9*	*171.3*	*123.6*	*95.3*	*74.3*	*63.4*	*50.0*	*43.0*	*33.2*	*29.8*	*23.5*	*21.9*
**14**	M	328.5	4968.4	9813.4	12853.1	14862.4	16206.7	17150.3	17835.0	18329.1	18701.5	18978.0	19189.5	19357.3	19483.7	19585.3
	SD	*296.9*	*827.0*	*344.3*	*204.1*	*149.8*	*113.2*	*87.8*	*68.6*	*53.6*	*50.3*	*38.0*	*33.3*	*29.6*	*26.3*	*22.8*
**16**	M	481.5	5950.8	10404.7	13208.1	15068.8	16338.1	17236.6	17880.3	18361.7	18717.2	18989.9	19197.8	19358.1	19486.8	19588.1
	SD	*415.5*	*614.5*	*305.1*	*207.1*	*139.2*	*109.8*	*83.8*	*68.9*	*54.1*	*44.8*	*36.6*	*31.8*	*30.1*	*24.6*	*22.0*
**18**	M	664.8	6611.4	10789.7	13448.2	15223.7	16425.9	17286.0	17918.3	18381.6	18728.6	18999.0	19201.6	19362.0	19488.7	19587.4
	SD	*565.7*	*503.2*	*276.0*	*182.8*	*133.6*	*99.6*	*82.2*	*62.6*	*55.2*	*46.0*	*38.9*	*32.4*	*28.8*	*24.5*	*21.8*
**20**	M	943.7	7100.8	11080.5	13626.9	15318.0	16491.0	17326.0	17940.6	18397.4	18736.2	19002.7	19208.4	19362.6	19489.9	19588.3
	SD	*735.0*	*447.4*	*274.6*	*170.6*	*129.3*	*95.9*	*79.1*	*68.2*	*52.7*	*45.8*	*38.8*	*35.1*	*28.7*	*23.7*	*21.8*

Mean values and standard deviation of $I_{tot,rep}$ **for** $4 \le n \le 20$**;** $1.2 \le \beta \cdot n \cdot \tau \le 4.0$**;** $\tau = 14$**;** $CC = 0.6$

n		$\beta \cdot n \cdot \tau$														
		**1.2**	**1.4**	**1.6**	**1.8**	**2.0**	**2.2**	**2.4**	**2.6**	**2.8**	**3.0**	**3.2**	**3.4**	**3.6**	**3.8**	**4.0**
**4**	M	37.4	50.7	64.2	82.3	98.7	121.9	152.5	183.8	218.8	268.4	319.2	379.3	440.5	528.6	647.3
	SD	*11.2*	*14.0*	*16.2*	*20.1*	*24.6*	*28.5*	*33.6*	*38.2*	*44.0*	*55.7*	*63.8*	*74.6*	*83.9*	*105.2*	*122.7*
**6**	M	52.8	78.0	117.3	173.9	281.7	562.8	1495.0	5644.0	10366.1	13491.3	15609.6	16946.0	17894.4	18544.9	18961.3
	SD	*18.6*	*24.2*	*36.2*	*62.5*	*112.0*	*265.1*	*810.9*	*1393.6*	*787.6*	*523.6*	*323.3*	*247.6*	*191.6*	*140.3*	*114.1*
**8**	M	65.8	102.7	169.8	327.6	882.2	4070.4	9432.9	12807.8	15152.9	16731.0	17765.4	18452.7	18897.4	19207.2	19426.8
	SD	*22.5*	*36.8*	*64.9*	*165.2*	*520.4*	*1413.4*	*756.6*	*464.8*	*345.0*	*242.2*	*190.6*	*138.0*	*105.3*	*84.1*	*55.9*
**10**	M	81.6	159.9	412.5	2271.0	7759.0	11632.5	14347.3	16107.4	17311.4	18119.6	18646.4	18997.0	19245.4	19425.0	19551.8
	SD	*33.2*	*71.6*	*249.9*	*1191.5*	*846.4*	*455.2*	*294.6*	*225.9*	*160.8*	*122.3*	*91.1*	*67.8*	*51.1*	*39.9*	*32.3*
**12**	M	96.9	248.2	1218.8	6718.2	11178.1	14044.7	15933.3	17142.7	17948.1	18492.3	18869.4	19129.5	19327.3	19471.5	19575.4
	SD	*44.3*	*143.1*	*794.5*	*734.6*	*415.9*	*289.8*	*196.4*	*139.5*	*103.9*	*74.6*	*57.6*	*47.1*	*32.1*	*28.8*	*23.9*
**14**	M	118.8	361.2	2984.7	9100.0	12838.6	15142.2	16624.1	17566.3	18195.5	18628.5	18942.4	19170.6	19346.4	19481.0	19585.2
	SD	*60.4*	*239.2*	*1294.5*	*553.5*	*324.4*	*202.3*	*142.7*	*105.9*	*75.8*	*59.5*	*46.9*	*35.2*	*31.0*	*26.3*	*22.8*
**16**	M	147.2	592.6	5475.4	10603.4	13728.5	15689.7	16916.7	17736.4	18284.2	18683.8	18970.3	19189.7	19353.4	19484.0	19583.4
	SD	*77.0*	*409.1*	*1051.0*	*400.5*	*283.0*	*174.6*	*121.6*	*85.7*	*63.3*	*52.0*	*39.5*	*35.5*	*29.5*	*25.4*	*22.5*
**18**	M	167.1	947.0	7032.0	11617.4	14300.1	16002.6	17097.1	17816.2	18338.9	18708.2	18986.4	19198.1	19362.2	19485.5	19586.4
	SD	*92.0*	*650.4*	*671.3*	*346.1*	*231.5*	*143.3*	*104.3*	*82.2*	*61.6*	*50.1*	*42.2*	*32.2*	*29.9*	*25.5*	*23.5*
**20**	M	203.9	1453.2	8124.9	12239.3	14691.6	16192.6	17200.8	17880.7	18367.6	18720.6	18994.7	19198.4	19363.7	19486.7	19587.4
	SD	*129.9*	*928.5*	*788.7*	*310.8*	*205.6*	*129.1*	*93.7*	*67.9*	*57.5*	*45.2*	*40.0*	*35.7*	*30.7*	*24.7*	*21.4*

Mean values and standard deviation of $I_{tot,ran}$ for $4 \le n \le 20$; $2 \le \tau \le 14$; $\beta \cdot n \cdot \tau = 1.6$

n		τ												
		**2**	**3**	**4**	**5**	**6**	**7**	**8**	**9**	**10**	**11**	**12**	**13**	**14**
**4**	M	12840.6	12818.1	12828.3	12847.1	12827.3	12825.9	12825.5	12829.8	12847.4	12827.5	12830.1	12822.6	12837.8
	SD	*146.3*	*155.9*	*159.7*	*166.1*	*165.1*	*149.1*	*159.9*	*158.8*	*149.1*	*156.0*	*165.4*	*155.6*	*148.8*
**6**	M	12836.5	12831.0	12844.3	12840.9	12828.4	12837.3	12830.8	12839.6	12824.8	12838.8	12835.3	12822.8	12839.5
	SD	*148.0*	*162.0*	*153.3*	*152.8*	*169.4*	*157.4*	*155.7*	*153.4*	*159.0*	*151.6*	*154.9*	*154.6*	*149.6*
**8**	M	12841.8	12824.4	12836.3	12847.1	12823.7	12836.0	12848.7	12839.5	12843.4	12838.9	12837.7	12835.4	12830.5
	SD	*163.4*	*157.0*	*157.3*	*155.1*	*151.9*	*157.0*	*151.4*	*159.2*	*165.6*	*157.0*	*148.1*	*158.0*	*160.9*
**10**	M	12834.9	12833.6	12837.7	12838.0	12837.9	12829.0	12830.2	12831.4	12846.8	12823.6	12822.9	12851.6	12823.0
	SD	*152.9*	*155.1*	*160.5*	*153.0*	*158.9*	*157.6*	*153.7*	*165.5*	*164.4*	*160.5*	*160.6*	*156.6*	*169.1*
**12**	M	12831.6	12829.9	12832.2	12830.6	12824.2	12830.0	12837.1	12833.0	12835.0	12839.2	12827.1	12831.7	12829.0
	SD	*153.4*	*151.9*	*151.8*	*175.1*	*146.2*	*159.7*	*162.1*	*153.3*	*160.2*	*159.2*	*164.9*	*157.9*	*164.1*
**14**	M	12825.5	12832.3	12836.0	12840.5	12828.7	12834.7	12839.1	12835.4	12832.3	12843.0	12830.4	12837.0	12833.5
	SD	*153.8*	*158.2*	*155.3*	*152.4*	*158.1*	*158.9*	*164.8*	*154.2*	*163.7*	*156.7*	*165.2*	*156.3*	*173.3*
**16**	M	12832.7	12839.1	12819.6	12832.6	12830.1	12844.5	12828.6	12835.2	12829.2	12828.4	12837.0	12837.7	12832.7
	SD	*156.9*	*163.8*	*152.7*	*160.1*	*151.5*	*157.7*	*156.3*	*153.9*	*161.8*	*153.9*	*168.0*	*160.8*	*162.3*
**18**	M	12844.8	12822.9	12830.0	12846.9	12834.9	12822.3	12852.3	12826.7	12837.7	12831.7	12831.2	12829.6	12839.5
	SD	*160.2*	*160.7*	*159.2*	*147.0*	*158.6*	*151.6*	*150.7*	*163.0*	*165.7*	*166.0*	*160.6*	*160.4*	*159.8*
**20**	M	12851.4	12839.7	12828.6	12834.9	12845.8	12839.4	12825.8	12839.5	12832.2	12841.3	12837.1	12837.4	12831.5
	SD	*149.4*	*156.4*	*155.3*	*156.2*	*156.7*	*162.2*	*167.7*	*170.0*	*167.6*	*158.2*	*167.2*	*159.1*	*163.8*

Mean values and standard deviation of $I_{tot,rep}$ **for** $4 \le n \le 20$ **;** $2 \le \tau \le 14$ **;** $\beta \cdot n \cdot \tau = 1.6$ **;** $CC = 0.0$

n							τ							
		**2**	**3**	**4**	**5**	**6**	**7**	**8**	**9**	**10**	**11**	**12**	**13**	**14**
**4**	M	5392.5	3323.1	2355.1	1791.5	1553.2	1356.9	1264.6	1178.9	1168.6	1031.5	1050.2	936.3	999.3
	SD	*956.4*	*1123.4*	*1241.4*	*1038.5*	*995.9*	*936.9*	*825.4*	*820.6*	*791.2*	*741.7*	*758.1*	*728.6*	*742.0*
**6**	M	10038.9	9306.8	8920.8	8702.4	8544.8	8458.8	8345.9	8324.0	8231.0	8237.8	8147.6	8129.3	8097.7
	SD	*289.1*	*332.2*	*344.8*	*364.5*	*585.7*	*347.3*	*399.6*	*561.9*	*585.2*	*393.9*	*381.3*	*715.0*	*383.6*
**8**	M	11149.2	10720.5	10505.1	10336.4	10255.2	10182.0	10145.3	10104.8	10080.5	10060.3	10042.8	10013.9	9982.7
	SD	*239.6*	*253.8*	*255.2*	*260.5*	*282.3*	*270.4*	*281.1*	*261.9*	*267.7*	*289.4*	*283.0*	*265.1*	*281.9*
**10**	M	11632.8	11314.3	11174.9	11069.4	10999.3	10946.8	10940.1	10895.0	10871.6	10808.5	10856.4	10834.6	10799.4
	SD	*225.7*	*223.3*	*231.5*	*249.0*	*231.9*	*249.0*	*232.5*	*238.1*	*245.1*	*623.8*	*230.4*	*246.5*	*250.2*
**12**	M	11905.7	11669.6	11539.0	11477.2	11423.3	11366.2	11348.7	11332.4	11323.8	11286.6	11274.0	11296.8	11249.1
	SD	*211.2*	*206.8*	*213.3*	*221.6*	*222.0*	*213.5*	*213.9*	*214.2*	*218.5*	*235.0*	*236.1*	*225.5*	*220.8*
**14**	M	12090.0	11887.5	11768.3	11718.0	11675.9	11650.3	11617.9	11596.8	11591.0	11585.8	11565.4	11568.5	11544.4
	SD	*187.6*	*207.3*	*202.4*	*211.4*	*191.2*	*204.9*	*212.9*	*204.6*	*212.3*	*209.4*	*202.3*	*197.4*	*225.5*
**16**	M	12193.2	12030.0	11937.2	11905.9	11858.9	11828.3	11815.6	11806.3	11787.8	11774.8	11758.8	11761.1	11754.7
	SD	*181.2*	*191.0*	*196.4*	*186.1*	*189.4*	*204.4*	*193.1*	*202.3*	*212.2*	*196.3*	*211.3*	*191.0*	*213.2*
**18**	M	12278.7	12146.7	12052.4	12016.6	11984.7	11984.4	11941.7	11941.1	11935.2	11913.1	11920.0	11914.0	11898.7
	SD	*179.3*	*193.2*	*188.1*	*191.0*	*198.9*	*183.7*	*191.7*	*196.1*	*200.0*	*204.6*	*189.5*	*204.8*	*196.3*
**20**	M	12359.1	12210.9	12145.3	12133.6	12085.6	12065.1	12067.3	12046.1	12032.7	12029.2	12031.3	12020.4	12012.0
	SD	*190.1*	*185.5*	*196.8*	*183.7*	*199.3*	*191.3*	*189.8*	*200.5*	*196.4*	*196.3*	*196.5*	*203.2*	*201.0*

Data tables for $I_{\max}$

Mean values and standard deviation of $I_{\max,ran}$ for $4 \le n \le 20$; $1.2 \le \beta \cdot n \cdot \tau \le 4.0$; $\tau = 14$

n		$\beta \cdot n \cdot \tau$														
		**1.2**	**1.4**	**1.6**	**1.8**	**2.0**	**2.2**	**2.4**	**2.6**	**2.8**	**3.0**	**3.2**	**3.4**	**3.6**	**3.8**	**4.0**
**4**	M	54.3	142.5	242.0	344.8	447.4	543.3	636.9	728.5	814.6	900.6	981.6	1058.8	1131.3	1207.0	1276.1
	SD	*8.1*	*10.3*	*11.7*	*13.6*	*15.1*	*15.4*	*17.2*	*18.0*	*19.8*	*21.5*	*23.8*	*22.9*	*22.7*	*24.0*	*25.5*
**6**	M	54.0	141.1	242.9	345.9	446.1	545.0	638.6	728.7	815.5	901.9	979.5	1058.5	1133.9	1205.1	1277.4
	SD	*9.0*	*9.6*	*11.2*	*13.0*	*14.5*	*15.5*	*16.8*	*19.2*	*20.2*	*20.5*	*20.1*	*22.3*	*21.9*	*26.6*	*26.3*
**8**	M	54.5	142.4	242.4	345.9	446.1	545.7	638.3	730.5	814.2	900.6	978.4	1056.5	1131.8	1208.5	1275.8
	SD	*9.3*	*9.7*	*11.2*	*13.1*	*14.9*	*14.9*	*17.8*	*18.9*	*20.4*	*21.9*	*20.1*	*22.8*	*22.7*	*26.7*	*26.0*
**10**	M	54.5	142.4	243.1	344.9	448.0	544.2	639.8	728.9	817.4	900.3	980.2	1057.5	1131.7	1206.5	1276.6
	SD	*9.3*	*10.2*	*11.4*	*13.7*	*14.2*	*16.8*	*16.6*	*17.6*	*20.1*	*20.0*	*23.3*	*23.7*	*23.2*	*23.7*	*25.0*
**12**	M	55.0	143.0	241.7	345.1	445.7	545.4	638.7	729.4	817.0	899.4	981.5	1059.1	1135.1	1205.8	1277.7
	SD	*8.2*	*10.5*	*11.6*	*13.2*	*14.6*	*16.9*	*15.2*	*18.7*	*19.8*	*20.4*	*21.9*	*21.9*	*23.8*	*25.4*	*25.2*
**14**	M	54.4	142.9	242.6	345.3	447.0	544.9	638.3	731.2	815.4	899.6	980.3	1058.4	1131.0	1205.1	1279.9
	SD	*8.5*	*10.6*	*12.2*	*13.2*	*14.1*	*15.9*	*16.9*	*18.7*	*19.4*	*20.3*	*23.1*	*22.7*	*22.9*	*23.8*	*27.0*
**16**	M	54.2	142.1	242.8	344.8	447.0	545.4	638.8	729.5	815.8	899.8	981.3	1058.0	1132.7	1205.8	1278.7
	SD	*8.7*	*10.8*	*11.1*	*14.4*	*15.1*	*16.1*	*17.2*	*18.8*	*17.7*	*21.6*	*20.8*	*23.8*	*24.0*	*24.2*	*26.7*
**18**	M	54.3	141.7	242.2	345.0	448.8	544.9	638.4	728.5	816.5	899.1	978.5	1059.6	1133.9	1205.9	1275.6
	SD	*9.6*	*10.6*	*12.1*	*13.7*	*15.2*	*15.0*	*16.8*	*17.7*	*21.0*	*19.9*	*20.6*	*20.3*	*24.1*	*25.4*	*25.7*
**20**	M	55.0	141.8	241.8	345.9	447.7	544.6	638.3	729.2	815.0	901.2	979.1	1057.4	1132.0	1205.7	1275.9
	SD	*10.2*	*10.8*	*11.0*	*13.4*	*15.1*	*15.7*	*16.9*	*19.8*	*20.4*	*20.4*	*20.9*	*23.8*	*22.7*	*24.3*	*25.5*

Mean values and standard deviation of $I_{\max,rep}$ **for** $4 \le n \le 20$ **;** $1.2 \le \beta \cdot n \cdot \tau \le 4.0$ **;** $\tau = 14$ **;** $CC = 0.0$

n		$\beta \cdot n \cdot \tau$														
		**1.2**	**1.4**	**1.6**	**1.8**	**2.0**	**2.2**	**2.4**	**2.6**	**2.8**	**3.0**	**3.2**	**3.4**	**3.6**	**3.8**	**4.0**
**4**	M	4.5	5.5	9.0	38.8	94.5	158.9	227.0	295.8	363.0	427.4	489.5	548.9	607.3	664.2	716.3
	SD	*1.2*	*1.4*	*3.2*	*7.6*	*8.8*	*11.0*	*11.1*	*11.9*	*12.9*	*14.9*	*15.2*	*16.1*	*15.5*	*16.0*	*17.5*
**6**	M	5.3	15.2	66.1	137.7	218.8	301.1	379.4	459.1	534.6	605.9	675.4	743.8	807.8	870.6	931.9
	SD	*1.4*	*5.8*	*8.1*	*9.6*	*11.7*	*12.5*	*12.9*	*14.7*	*15.6*	*16.3*	*17.6*	*19.7*	*19.3*	*20.0*	*21.7*
**8**	M	7.0	39.0	108.1	193.0	280.1	365.9	450.3	533.2	610.5	689.1	760.0	831.2	897.2	965.3	1028.3
	SD	*2.7*	*7.7*	*9.5*	*11.1*	*12.1*	*12.7*	*14.7*	*15.6*	*16.8*	*18.7*	*19.0*	*18.7*	*21.1*	*21.8*	*21.1*
**10**	M	9.9	56.9	135.9	223.4	313.6	404.4	491.9	576.0	655.9	733.4	808.7	879.1	949.9	1014.5	1084.2
	SD	*4.5*	*8.6*	*9.9*	*11.4*	*12.0*	*14.7*	*16.1*	*16.4*	*18.6*	*18.5*	*19.2*	*20.9*	*19.9*	*22.0*	*24.2*
**12**	M	13.4	70.5	153.9	244.9	338.3	428.4	517.0	602.2	685.1	763.8	836.4	911.7	982.0	1049.7	1117.3
	SD	*5.7*	*8.8*	*9.8*	*11.6*	*13.4*	*14.3*	*16.9*	*16.9*	*19.7*	*18.2*	*19.6*	*20.5*	*21.7*	*22.0*	*22.6*
**14**	M	17.7	80.5	166.1	259.8	354.3	446.7	536.8	621.9	704.4	783.1	860.3	934.7	1004.3	1073.8	1142.5
	SD	*6.6*	*8.6*	*9.9*	*12.4*	*12.5*	*14.1*	*16.5*	*16.7*	*18.6*	*20.6*	*20.0*	*20.7*	*22.8*	*21.7*	*25.5*
**16**	M	21.0	88.1	175.9	270.6	364.9	459.4	549.5	635.7	719.0	799.1	874.9	950.8	1022.0	1091.6	1160.0
	SD	*7.0*	*8.7*	*11.0*	*12.7*	*13.7*	*14.8*	*15.7*	*18.0*	*19.1*	*19.9*	*20.2*	*19.1*	*22.6*	*23.9*	*24.9*
**18**	M	24.2	93.9	183.4	279.9	375.5	468.8	559.4	647.4	731.0	810.4	887.9	962.8	1032.9	1103.3	1171.9
	SD	*7.9*	*9.1*	*10.8*	*13.4*	*14.0*	*15.8*	*16.4*	*17.1*	*18.9*	*18.2*	*19.8*	*22.4*	*21.3*	*22.9*	*24.0*
**20**	M	27.4	98.6	188.9	287.0	382.0	476.6	569.4	654.8	739.3	817.6	898.3	972.1	1044.3	1115.2	1184.5
	SD	*7.7*	*9.2*	*10.9*	*11.7*	*14.6*	*13.2*	*16.1*	*17.1*	*18.2*	*18.9*	*21.3*	*21.4*	*21.9*	*24.3*	*23.9*

Mean values and standard deviation of $I_{\max,rep}$ **for** $4 \le n \le 20$ **;** $1.2 \le \beta \cdot n \cdot \tau \le 4.0$ **;** $\tau = 14$ **;** $CC = 0.2$

n		$\beta \cdot n \cdot \tau$														
		**1.2**	**1.4**	**1.6**	**1.8**	**2.0**	**2.2**	**2.4**	**2.6**	**2.8**	**3.0**	**3.2**	**3.4**	**3.6**	**3.8**	**4.0**
**4**	M	4.3	5.1	6.2	9.8	34.2	80.9	136.0	193.0	252.3	308.0	363.8	415.1	466.9	515.6	563.0
	SD	*1.1*	*1.1*	*1.4*	*3.1*	*7.2*	*8.3*	*9.6*	*11.4*	*11.4*	*12.7*	*12.6*	*14.0*	*13.5*	*15.6*	*14.5*
**6**	M	4.9	7.1	27.2	81.9	150.3	223.9	296.7	368.5	438.4	504.7	568.4	631.4	689.8	744.4	800.5
	SD	*1.2*	*2.1*	*6.9*	*8.5*	*10.3*	*11.2*	*11.5*	*12.6*	*13.7*	*15.0*	*15.8*	*16.1*	*16.8*	*18.4*	*19.3*
**8**	M	5.7	17.5	71.0	144.5	225.8	305.9	387.4	462.3	536.7	608.7	676.3	740.4	806.9	867.3	927.0
	SD	*1.7*	*6.2*	*9.2*	*9.4*	*11.1*	*12.9*	*13.5*	*15.4*	*16.2*	*16.8*	*16.8*	*17.9*	*19.0*	*20.2*	*20.9*
**10**	M	6.9	34.5	101.7	183.5	268.3	353.7	437.1	515.7	592.4	667.0	736.7	804.2	871.2	934.9	994.0
	SD	*2.5*	*7.7*	*9.4*	*11.0*	*12.1*	*13.5*	*13.8*	*15.8*	*17.7*	*17.6*	*18.4*	*20.2*	*21.1*	*20.2*	*22.3*
**12**	M	8.5	49.0	124.5	208.9	297.5	386.3	470.8	551.5	628.2	704.1	777.2	845.3	913.9	980.6	1042.3
	SD	*3.6*	*8.1*	*9.9*	*10.9*	*13.4*	*13.0*	*16.1*	*15.7*	*17.9*	*17.6*	*20.1*	*20.3*	*21.0*	*21.2*	*23.3*
**14**	M	10.8	60.3	139.6	228.8	319.2	407.0	494.2	576.3	655.7	732.0	804.3	874.2	943.6	1009.8	1072.9
	SD	*4.4*	*8.4*	*9.9*	*12.0*	*13.6*	*13.6*	*15.0*	*16.4*	*17.3*	*19.5*	*19.6*	*19.8*	*21.6*	*22.6*	*24.5*
**16**	M	13.1	69.6	151.6	241.6	334.3	424.2	511.3	594.9	675.3	751.2	825.8	898.5	965.9	1032.1	1099.2
	SD	*5.6*	*8.6*	*10.5*	*11.7*	*13.6*	*14.3*	*15.0*	*16.6*	*17.5*	*17.6*	*20.2*	*20.6*	*23.2*	*23.1*	*24.2*
**18**	M	15.5	76.5	160.5	253.3	345.3	436.8	524.5	608.0	690.8	767.3	842.9	913.1	983.9	1051.3	1117.4
	SD	*6.3*	*9.3*	*10.6*	*12.5*	*12.8*	*13.7*	*14.3*	*17.1*	*18.2*	*18.5*	*20.0*	*20.7*	*22.4*	*23.6*	*24.1*
**20**	M	18.4	82.3	168.3	262.4	356.1	447.3	536.2	621.0	701.7	780.1	856.2	926.7	996.7	1064.4	1131.5
	SD	*6.8*	*8.4*	*10.7*	*12.2*	*13.7*	*14.8*	*15.8*	*17.6*	*17.8*	*20.0*	*20.2*	*19.9*	*21.6*	*25.4*	*23.4*

Mean values and standard deviation of $I_{\max,rep}$ for $4 \le n \le 20$; $1.2 \le \beta \cdot n \cdot \tau \le 4.0$; $\tau = 14$; $CC = 0.4$

n		$\beta \cdot n \cdot \tau$														
		**1.2**	**1.4**	**1.6**	**1.8**	**2.0**	**2.2**	**2.4**	**2.6**	**2.8**	**3.0**	**3.2**	**3.4**	**3.6**	**3.8**	**4.0**
**4**	M	4.1	4.8	5.2	6.2	7.1	8.7	15.5	37.8	69.1	105.1	142.0	179.0	215.6	250.6	285.2
	SD	*1.1*	*1.3*	*1.3*	*1.4*	*1.5*	*1.9*	*4.8*	*7.6*	*8.4*	*9.0*	*9.7*	*10.4*	*11.8*	*11.4*	*11.8*
**6**	M	4.6	5.5	7.4	18.6	58.9	111.6	171.3	231.9	289.3	345.4	399.9	452.0	501.3	548.3	593.0
	SD	*1.2*	*1.4*	*1.8*	*6.0*	*8.2*	*9.3*	*10.2*	*12.3*	*12.7*	*11.8*	*13.0*	*14.5*	*16.1*	*15.4*	*17.3*
**8**	M	5.1	6.9	22.6	72.7	138.2	208.6	278.0	346.4	411.4	474.0	534.0	593.5	647.5	700.6	752.2
	SD	*1.3*	*1.9*	*6.4*	*8.8*	*9.5*	*11.7*	*11.9*	*14.2*	*14.5*	*14.7*	*16.0*	*17.3*	*18.6*	*18.7*	*20.1*
**10**	M	5.5	10.7	50.9	115.9	191.0	266.7	343.3	414.3	485.1	550.8	614.1	672.0	732.7	789.0	843.9
	SD	*1.6*	*4.1*	*7.7*	*9.8*	*10.5*	*12.1*	*14.0*	*14.6*	*16.3*	*15.9*	*16.8*	*17.8*	*19.2*	*19.1*	*19.0*
**12**	M	5.8	18.8	74.3	149.1	227.9	307.5	384.3	458.3	532.4	598.2	663.1	725.7	786.7	846.5	902.8
	SD	*1.7*	*6.2*	*8.4*	*10.5*	*11.6*	*12.9*	*13.1*	*15.0*	*16.6*	*16.3*	*18.8*	*19.5*	*18.9*	*20.4*	*19.8*
**14**	M	6.4	29.2	91.9	171.2	253.8	336.3	415.4	492.4	564.2	635.0	699.6	764.5	825.8	884.7	942.2
	SD	*2.1*	*7.2*	*8.9*	*10.3*	*12.1*	*13.9*	*14.1*	*14.9*	*16.7*	*18.4*	*17.8*	*18.9*	*19.9*	*19.8*	*22.6*
**16**	M	7.3	38.7	107.6	188.5	273.7	357.8	438.2	514.7	589.7	659.0	728.0	792.3	856.5	916.9	975.6
	SD	*2.6*	*7.5*	*10.2*	*10.8*	*12.5*	*13.5*	*14.1*	*15.8*	*16.7*	*18.3*	*17.8*	*19.8*	*20.1*	*21.0*	*23.3*
**18**	M	8.3	46.4	119.4	204.1	290.4	373.8	455.7	534.1	608.4	680.3	749.7	815.7	879.1	939.9	999.6
	SD	*3.1*	*8.3*	*10.3*	*11.0*	*13.1*	*13.8*	*14.8*	*15.7*	*17.2*	*17.2*	*20.0*	*20.5*	*19.9*	*20.6*	*22.7*
**20**	M	9.3	53.8	130.3	215.8	304.3	388.7	471.3	548.9	624.5	697.7	768.4	833.1	897.4	961.4	1020.0
	SD	*3.6*	*8.2*	*10.0*	*12.0*	*13.6*	*14.8*	*15.3*	*16.0*	*16.0*	*17.9*	*18.4*	*18.7*	*21.2*	*21.4*	*21.7*

Mean values and standard deviation of $I_{\max,rep}$ for $4 \le n \le 20$; $1.2 \le \beta \cdot n \cdot \tau \le 4.0$; $\tau = 14$; $CC = 0.6$

n		$\beta \cdot n \cdot \tau$														
		**1.2**	**1.4**	**1.6**	**1.8**	**2.0**	**2.2**	**2.4**	**2.6**	**2.8**	**3.0**	**3.2**	**3.4**	**3.6**	**3.8**	**4.0**
**4**	M	4.1	4.6	5.3	5.8	6.2	6.8	7.5	8.0	8.6	9.3	10.0	10.3	10.9	11.7	12.3
	SD	*1.0*	*1.1*	*1.2*	*1.2*	*1.3*	*1.3*	*1.5*	*1.4*	*1.5*	*1.6*	*1.6*	*1.6*	*1.6*	*1.5*	*1.7*
**6**	M	4.4	5.2	6.2	6.9	7.9	9.6	12.0	23.1	47.3	77.9	110.8	142.3	173.5	203.5	230.9
	SD	*1.2*	*1.2*	*1.4*	*1.5*	*1.6*	*1.9*	*2.9*	*5.9*	*7.7*	*9.1*	*9.1*	*10.5*	*11.4*	*11.5*	*11.4*
**8**	M	4.8	5.6	6.7	8.1	10.5	19.6	47.1	82.3	121.2	162.1	200.5	236.3	269.3	302.1	334.1
	SD	*1.1*	*1.4*	*1.5*	*1.8*	*2.5*	*5.4*	*7.7*	*9.3*	*10.4*	*12.1*	*12.5*	*12.7*	*12.5*	*13.6*	*13.7*
**10**	M	5.0	6.4	7.9	14.9	43.2	87.9	137.6	188.5	236.7	285.2	329.3	370.3	411.8	451.9	486.3
	SD	*1.3*	*1.5*	*2.0*	*4.9*	*8.1*	*9.6*	*9.9*	*11.5*	*12.3*	*13.1*	*14.4*	*15.2*	*14.2*	*16.0*	*16.6*
**12**	M	5.2	6.9	11.1	36.6	85.8	142.9	199.8	256.5	307.8	360.4	409.6	454.2	501.2	544.0	586.5
	SD	*1.4*	*1.7*	*3.5*	*7.0*	*9.1*	*11.4*	*11.3*	*12.7*	*14.2*	*14.0*	*14.7*	*15.8*	*17.0*	*16.8*	*18.4*
**14**	M	5.4	7.5	17.0	58.7	116.1	178.2	241.4	299.7	356.4	410.6	461.7	512.0	558.6	602.4	646.5
	SD	*1.5*	*1.9*	*5.7*	*8.4*	*10.4*	*11.3*	*12.7*	*13.8*	*14.6*	*15.3*	*16.8*	*17.9*	*17.7*	*18.7*	*19.2*
**16**	M	5.7	8.5	28.6	80.2	143.5	210.2	274.8	336.5	395.2	453.9	504.3	556.2	603.2	649.5	697.9
	SD	*1.6*	*2.5*	*7.4*	*9.2*	*10.0*	*11.5*	*13.5*	*14.5*	*15.0*	*15.8*	*16.3*	*18.6*	*17.1*	*19.8*	*20.6*
**18**	M	6.0	9.8	39.8	98.8	166.7	234.5	300.6	364.7	425.6	481.3	538.2	589.1	639.5	688.4	734.8
	SD	*1.7*	*3.0*	*7.6*	*9.8*	*11.5*	*13.3*	*13.6*	*14.1*	*16.8*	*16.6*	*16.6*	*18.8*	*18.6*	*20.6*	*22.6*
**20**	M	6.1	11.7	51.2	114.7	185.6	255.4	322.8	388.7	450.0	508.2	564.7	618.5	669.9	717.7	766.3
	SD	*1.6*	*4.0*	*8.7*	*9.5*	*11.7*	*12.1*	*13.9*	*15.0*	*16.5*	*17.0*	*17.3*	*18.6*	*19.1*	*20.2*	*21.6*

Data tables for $t_{\mathrm{Im}ax,ran}$

Mean values and standard deviation of the time to peak $t_{\mathrm{Im}ax,ran}$ for $4 \le n \le 20$; $1.2 \le \beta \cdot n \cdot \tau \le 4.0$; $\tau = 14$

n		$\beta \cdot n \cdot \tau$														
		**1.2**	**1.4**	**1.6**	**1.8**	**2.0**	**2.2**	**2.4**	**2.6**	**2.8**	**3.0**	**3.2**	**3.4**	**3.6**	**3.8**	**4.0**
**4**	M	176.6	114.4	87.7	72.7	62.7	55.6	49.5	45.4	41.9	38.8	36.6	34.4	32.6	31.1	29.5
	SD	*37.0*	*12.2*	*6.7*	*4.9*	*3.8*	*3.0*	*2.6*	*2.4*	*2.0*	*1.7*	*1.6*	*1.5*	*1.4*	*1.3*	*1.2*
**6**	M	176.1	114.8	88.1	72.6	62.4	55.3	49.7	45.3	41.9	39.1	36.5	34.5	32.7	31.0	29.5
	SD	*38.4*	*12.1*	*7.6*	*4.8*	*3.7*	*3.0*	*2.5*	*2.1*	*2.0*	*1.8*	*1.7*	*1.5*	*1.5*	*1.3*	*1.2*
**8**	M	174.2	114.3	88.5	72.6	62.6	55.3	49.9	45.4	41.8	38.8	36.6	34.4	32.6	30.8	29.4
	SD	*38.7*	*12.5*	*7.1*	*5.1*	*3.8*	*3.1*	*2.5*	*2.3*	*2.0*	*1.7*	*1.6*	*1.6*	*1.3*	*1.3*	*1.2*
**10**	M	179.0	114.6	88.1	72.7	62.4	55.2	49.6	45.3	42.0	39.2	36.5	34.4	32.4	30.9	29.3
	SD	*40.4*	*11.9*	*7.1*	*4.8*	*3.7*	*3.2*	*2.6*	*2.4*	*2.0*	*1.9*	*1.7*	*1.5*	*1.3*	*1.3*	*1.2*
**12**	M	175.4	114.4	88.6	73.0	62.5	55.5	49.7	45.8	41.9	39.0	36.5	34.4	32.6	30.8	29.5
	SD	*34.8*	*12.4*	*7.0*	*5.4*	*3.7*	*3.1*	*2.6*	*2.3*	*2.0*	*1.8*	*1.6*	*1.5*	*1.4*	*1.2*	*1.2*
**14**	M	173.3	116.3	88.1	72.8	62.7	55.1	49.8	45.5	41.8	38.9	36.6	34.5	32.6	30.9	29.4
	SD	*36.3*	*14.0*	*6.7*	*5.1*	*3.6*	*3.1*	*2.6*	*2.2*	*2.0*	*1.9*	*1.7*	*1.6*	*1.4*	*1.3*	*1.2*
**16**	M	174.5	115.0	88.3	73.0	62.8	55.5	49.5	45.4	41.9	39.0	36.5	34.5	32.5	31.0	29.5
	SD	*38.0*	*12.1*	*6.5*	*5.1*	*3.6*	*3.1*	*2.6*	*2.4*	*2.0*	*1.9*	*1.6*	*1.5*	*1.4*	*1.4*	*1.3*
**18**	M	172.4	115.9	88.2	72.6	62.6	55.0	49.6	45.3	41.9	38.8	36.5	34.3	32.6	30.9	29.4
	SD	*38.5*	*14.5*	*7.0*	*4.8*	*3.7*	*3.2*	*2.5*	*2.2*	*1.9*	*1.9*	*1.7*	*1.5*	*1.4*	*1.3*	*1.3*
**20**	M	172.7	115.7	88.3	72.8	62.4	55.5	49.7	45.3	41.8	39.0	36.6	34.3	32.5	31.1	29.5
	SD	*42.9*	*12.8*	*6.9*	*4.8*	*3.6*	*3.0*	*2.5*	*2.2*	*1.9*	*1.7*	*1.7*	*1.5*	*1.4*	*1.4*	*1.2*

Mean values and standard deviation of time to peak $t_{\mathrm{Im}ax,rep}$ **for** $4 \le n \le 20$ **;** $1.2 \le \beta \cdot n \cdot \tau \le 4.0$ **;** $\tau = 14$ **;** $CC = 0.0$

n		$\beta \cdot n \cdot \tau$														
		**1.2**	**1.4**	**1.6**	**1.8**	**2.0**	**2.2**	**2.4**	**2.6**	**2.8**	**3.0**	**3.2**	**3.4**	**3.6**	**3.8**	**4.0**
**4**	M	12.0	19.4	113.8	251.6	170.1	131.5	109.0	93.4	83.4	75.2	68.5	63.0	58.4	54.8	52.0
	SD	*10.4*	*22.0*	*123.6*	*74.0*	*21.1*	*11.4*	*7.9*	*6.2*	*4.7*	*3.8*	*3.6*	*3.0*	*2.5*	*2.4*	*2.2*
**6**	M	23.4	229.1	180.0	131.0	103.8	87.3	75.7	67.7	61.1	56.1	51.9	48.4	54.4	42.8	40.6
	SD	*29.9*	*176.2*	*33.0*	*13.4*	*8.2*	*5.9*	*4.3*	*3.6*	*3.1*	*2.5*	*2.4*	*2.1*	*1.9*	*1.9*	*1.7*
**8**	M	67.8	225.1	142.0	107.3	87.9	75.4	66.5	59.6	54.2	50.1	46.8	43.6	41.1	39.0	37.0
	SD	*91.8*	*62.7*	*18.5*	*9.7*	*6.3*	*4.8*	*4.0*	*3.2*	*2.7*	*2.3*	*2.1*	*1.9*	*1.6*	*1.5*	*1.4*
**10**	M	139.8	184.7	125.0	97.4	81.2	70.3	61.9	56.1	51.2	47.3	44.2	41.3	39.0	36.9	34.9
	SD	*133.2*	*35.2*	*12.9*	*7.4*	*5.5*	*4.2*	*3.5*	*3.8*	*2.6*	*2.2*	*1.9*	*1.9*	*1.6*	*1.6*	*1.5*
**12**	M	216.5	166.8	117.1	92.2	77.1	67.1	59.3	53.9	49.4	45.4	42.6	39.9	37.8	35.7	33.9
	SD	*156.6*	*24.9*	*11.6*	*6.8*	*4.7*	*3.8*	*3.3*	*2.9*	*2.4*	*2.1*	*1.8*	*1.8*	*1.7*	*1.5*	*1.3*
**14**	M	227.6	158.1	111.2	88.8	74.7	64.9	57.9	52.4	47.9	44.7	41.5	39.0	37.0	35.0	33.2
	SD	*127.4*	*28.4*	*10.5*	*6.8*	*5.1*	*3.8*	*3.0*	*2.8*	*2.4*	*2.2*	*1.9*	*1.7*	*1.6*	*1.5*	*1.3*
**16**	M	243.0	150.3	107.5	86.2	73.0	63.4	56.5	51.4	47.2	43.7	40.9	38.3	36.1	34.4	32.7
	SD	*124.8*	*22.1*	*10.2*	*6.3*	*4.7*	*3.9*	*3.0*	*2.4*	*2.3*	*2.0*	*1.8*	*1.7*	*1.5*	*1.5*	*1.4*
**18**	M	236.6	146.5	104.9	84.9	71.4	62.6	55.7	50.6	46.5	43.1	40.3	37.9	35.6	33.9	32.2
	SD	*104.0*	*20.9*	*9.4*	*6.1*	*4.5*	*3.5*	*3.0*	*2.6*	*2.3*	*2.0*	*1.8*	*1.7*	*1.4*	*1.5*	*1.4*
**20**	M	238.2	142.1	103.1	83.5	70.7	61.7	55.2	49.9	45.9	42.6	39.8	37.6	35.5	33.6	31.9
	SD	*94.8*	*19.0*	*8.9*	*6.2*	*4.7*	*3.8*	*3.0*	*2.5*	*2.3*	*2.0*	*1.9*	*1.7*	*1.5*	*1.4*	*1.3*

Mean values and standard deviation of time to peak $t_{\mathrm{Im}ax,rep}$ for $4 \le n \le 20$; $1.2 \le \beta \cdot n \cdot \tau \le 4.0$; $\tau = 14$; $CC = 0.2$

n		$\beta \cdot n \cdot \tau$														
		**1.2**	**1.4**	**1.6**	**1.8**	**2.0**	**2.2**	**2.4**	**2.6**	**2.8**	**3.0**	**3.2**	**3.4**	**3.6**	**3.8**	**4.0**
**4**	M	9.8	12.6	20.7	122.1	265.8	185.2	147.1	121.9	106.0	93.5	84.5	77.4	71.3	66.6	62.6
	SD	*7.4*	*10.0*	*26.2*	*138.1*	*96.8*	*27.4*	*14.2*	*10.2*	*7.7*	*5.8*	*4.8*	*3.7*	*3.6*	*3.4*	*3.0*
**6**	M	16.6	51.2	260.9	172.6	132.0	107.0	91.5	80.5	72.3	65.8	60.3	56.1	52.8	49.4	46.9
	SD	*20.7*	*69.7*	*105.1*	*23.7*	*13.1*	*8.0*	*5.8*	*4.5*	*4.1*	*3.6*	*2.9*	*2.6*	*2.4*	*2.1*	*2.0*
**8**	M	27.3	250.2	176.3	129.0	102.4	86.6	75.5	67.7	61.2	56.1	51.9	48.4	45.5	42.9	40.9
	SD	*39.1*	*158.7*	*29.7*	*13.2*	*8.4*	*5.9*	*4.4*	*3.9*	*3.2*	*2.7*	*2.4*	*2.1*	*1.9*	*1.6*	*1.6*
**10**	M	60.1	229.3	148.3	111.3	90.9	77.9	69.1	61.7	56.3	51.7	48.0	45.0	42.3	40.0	38.2
	SD	*75.0*	*74.8*	*19.3*	*9.3*	*6.8*	*5.2*	*4.1*	*3.4*	*2.9*	*2.5*	*2.1*	*1.9*	*1.9*	*1.7*	*1.5*
**12**	M	96.9	198.7	133.8	102.6	84.9	73.2	64.7	58.2	53.2	49.2	45.9	43.0	40.4	38.2	36.4
	SD	*108.7*	*42.4*	*14.6*	*8.4*	*5.9*	*4.2*	*3.5*	*3.1*	*2.7*	*2.4*	*2.2*	*1.9*	*1.6*	*1.6*	*1.5*
**14**	M	150.3	181.6	123.2	97.0	81.3	70.5	62.0	56.2	51.3	47.5	44.4	41.6	39.2	37.2	35.4
	SD	*132.1*	*35.9*	*13.6*	*7.7*	*5.9*	*4.4*	*3.5*	*3.1*	*2.7*	*2.2*	*2.1*	*1.7*	*1.6*	*1.6*	*1.4*
**16**	M	196.3	168.0	119.6	93.9	78.0	68.1	60.5	54.6	50.3	46.3	43.3	40.6	38.4	36.2	34.5
	SD	*147.5*	*31.7*	*12.4*	*7.3*	*5.3*	*3.9*	*3.2*	*2.8*	*2.5*	*2.3*	*2.0*	*1.7*	*1.7*	*1.5*	*1.4*
**18**	M	224.5	160.9	114.6	90.7	76.3	66.3	59.2	53.5	49.3	45.4	42.6	39.9	37.6	35.7	34.0
	SD	*148.6*	*25.7*	*10.8*	*6.9*	*5.0*	*4.0*	*3.0*	*2.8*	*2.5*	*2.0*	*2.0*	*1.8*	*1.6*	*1.6*	*1.4*
**20**	M	258.8	156.6	114.4	88.7	75.2	65.6	58.2	52.6	48.4	45.0	41.9	39.4	37.3	35.2	33.4
	SD	*158.7*	*25.3*	*11.3*	*6.7*	*4.6*	*3.9*	*3.2*	*2.8*	*2.2*	*2.1*	*1.9*	*1.7*	*1.5*	*1.4*	*1.3*

Mean values and standard deviation of time to peak $t_{\mathrm{Im}ax,rep}$ for $4 \le n \le 20$; $1.2 \le \beta \cdot n \cdot \tau \le 4.0$; $\tau = 14$; $CC = 0.4$

n		$\beta \cdot n \cdot \tau$														
		**1.2**	**1.4**	**1.6**	**1.8**	**2.0**	**2.2**	**2.4**	**2.6**	**2.8**	**3.0**	**3.2**	**3.4**	**3.6**	**3.8**	**4.0**
**4**	M	10.0	13.2	21.0	137.2	261.4	184.5	144.7	122.5	105.3	93.8	84.5	77.1	71.7	66.5	62.6
	SD	*6.8*	*13.6*	*27.6*	*151.7*	*84.8*	*25.4*	*14.3*	*9.3*	*7.2*	*6.1*	*4.8*	*3.9*	*3.9*	*3.2*	*3.1*
**6**	M	15.4	54.7	265.3	175.2	131.6	106.9	90.9	80.4	72.2	65.6	60.6	56.2	52.5	49.6	46.8
	SD	*19.2*	*73.8*	*103.8*	*26.4*	*12.1*	*8.7*	*6.1*	*4.9*	*3.7*	*3.3*	*2.7*	*2.4*	*2.3*	*2.1*	*1.9*
**8**	M	26.5	238.9	178.4	127.1	103.0	86.8	76.0	67.4	61.0	56.0	52.1	48.7	45.5	43.1	40.8
	SD	*30.5*	*151.5*	*30.8*	*12.5*	*8.5*	*5.6*	*4.6*	*3.8*	*3.2*	*2.6*	*2.3*	*2.2*	*2.1*	*1.9*	*1.8*
**10**	M	61.1	234.1	146.7	112.0	90.9	77.9	68.5	62.0	56.0	51.7	48.2	45.1	42.4	40.1	38.1
	SD	*86.0*	*71.7*	*18.7*	*9.5*	*6.5*	*4.8*	*3.8*	*3.4*	*2.8*	*2.3*	*2.2*	*2.0*	*1.8*	*1.7*	*1.7*
**12**	M	99.4	201.1	133.0	103.4	85.4	72.9	64.8	58.3	53.3	49.1	45.9	43.0	40.4	38.2	36.4
	SD	*111.4*	*42.0*	*14.5*	*8.9*	*6.0*	*4.7*	*3.5*	*3.2*	*2.7*	*2.4*	*2.2*	*1.8*	*1.7*	*1.5*	*1.5*
**14**	M	157.3	182.3	124.3	96.9	81.2	70.5	62.3	56.2	51.5	47.5	44.2	41.5	39.2	37.1	35.4
	SD	*137.3*	*32.0*	*13.0*	*8.0*	*5.8*	*4.4*	*3.5*	*2.9*	*2.5*	*2.3*	*1.9*	*1.8*	*1.6*	*1.5*	*1.4*
**16**	M	189.3	171.4	118.9	93.8	78.8	68.3	60.7	55.0	50.3	46.4	43.3	40.5	38.4	36.3	34.6
	SD	*140.5*	*31.8*	*12.6*	*7.3*	*5.7*	*4.0*	*3.5*	*2.9*	*2.5*	*2.2*	*2.0*	*1.8*	*1.7*	*1.6*	*1.4*
**18**	M	217.8	163.3	114.6	91.3	76.2	66.5	59.2	53.6	49.1	45.6	42.5	39.8	37.6	35.8	33.8
	SD	*140.7*	*26.8*	*11.5*	*7.0*	*5.0*	*3.7*	*3.2*	*2.7*	*2.3*	*2.3*	*2.0*	*1.7*	*1.6*	*1.5*	*1.4*
**20**	M	253.6	157.8	111.7	88.6	74.9	65.5	58.4	52.8	48.5	44.8	41.9	39.5	37.2	35.2	33.5
	SD	*151.9*	*24.3*	*10.9*	*6.7*	*5.1*	*3.7*	*3.1*	*2.9*	*2.5*	*2.0*	*2.0*	*1.8*	*1.6*	*1.5*	*1.5*

Mean values and standard deviation of time to peak $t_{\mathrm{Im}ax,rep}$ for $4 \le n \le 20$; $1.2 \le \beta \cdot n \cdot \tau \le 4.0$; $\tau = 14$; $CC = 0.6$

n		$\beta \cdot n \cdot \tau$														
		**1.2**	**1.4**	**1.6**	**1.8**	**2.0**	**2.2**	**2.4**	**2.6**	**2.8**	**3.0**	**3.2**	**3.4**	**3.6**	**3.8**	**4.0**
**4**	M	10.0	13.2	21.0	137.2	261.4	184.5	144.7	122.5	105.3	93.8	84.5	77.1	71.7	66.5	62.6
	SD	*6.8*	*13.6*	*27.6*	*151.7*	*84.8*	*25.4*	*14.3*	*9.3*	*7.2*	*6.1*	*4.8*	*3.9*	*3.9*	*3.2*	*3.1*
**6**	M	15.4	54.7	265.3	175.2	131.6	106.9	90.9	80.4	72.2	65.6	60.6	56.2	52.5	49.6	46.8
	SD	*19.2*	*73.8*	*103.8*	*26.4*	*12.1*	*8.7*	*6.1*	*4.9*	*3.7*	*3.3*	*2.7*	*2.4*	*2.3*	*2.1*	*1.9*
**8**	M	26.5	238.9	178.4	127.1	103.0	86.8	76.0	67.4	61.0	56.0	52.1	48.7	45.5	43.1	40.8
	SD	*30.5*	*151.5*	*30.8*	*12.5*	*8.5*	*5.6*	*4.6*	*3.8*	*3.2*	*2.6*	*2.3*	*2.2*	*2.1*	*1.9*	*1.8*
**10**	M	61.1	234.1	146.7	112.0	90.9	77.9	68.5	62.0	56.0	51.7	48.2	45.1	42.4	40.1	38.1
	SD	*86.0*	*71.7*	*18.7*	*9.5*	*6.5*	*4.8*	*3.8*	*3.4*	*2.8*	*2.3*	*2.2*	*2.0*	*1.8*	*1.7*	*1.7*
**12**	M	99.4	201.1	133.0	103.4	85.4	72.9	64.8	58.3	53.3	49.1	45.9	43.0	40.4	38.2	36.4
	SD	*111.4*	*42.0*	*14.5*	*8.9*	*6.0*	*4.7*	*3.5*	*3.2*	*2.7*	*2.4*	*2.2*	*1.8*	*1.7*	*1.5*	*1.5*
**14**	M	157.3	182.3	124.3	96.9	81.2	70.5	62.3	56.2	51.5	47.5	44.2	41.5	39.2	37.1	35.4
	SD	*137.3*	*32.0*	*13.0*	*8.0*	*5.8*	*4.4*	*3.5*	*2.9*	*2.5*	*2.3*	*1.9*	*1.8*	*1.6*	*1.5*	*1.4*
**16**	M	189.3	171.4	118.9	93.8	78.8	68.3	60.7	55.0	50.3	46.4	43.3	40.5	38.4	36.3	34.6
	SD	*140.5*	*31.8*	*12.6*	*7.3*	*5.7*	*4.0*	*3.5*	*2.9*	*2.5*	*2.2*	*2.0*	*1.8*	*1.7*	*1.6*	*1.4*
**18**	M	217.8	163.3	114.6	91.3	76.2	66.5	59.2	53.6	49.1	45.6	42.5	39.8	37.6	35.8	33.8
	SD	*140.7*	*26.8*	*11.5*	*7.0*	*5.0*	*3.7*	*3.2*	*2.7*	*2.3*	*2.3*	*2.0*	*1.7*	*1.6*	*1.5*	*1.4*
**20**	M	253.6	157.8	111.7	88.6	74.9	65.5	58.4	52.8	48.5	44.8	41.9	39.5	37.2	35.2	33.5
	SD	*151.9*	*24.3*	*10.9*	*6.7*	*5.1*	*3.7*	*3.1*	*2.9*	*2.5*	*2.0*	*2.0*	*1.8*	*1.6*	*1.5*	*1.5*
