# Supplementary material for: Models of epidemics: when contact repetition and clustering should be included
Source: Theor Biol Med Model. 2009 Jun 29;6:11. doi: 10.1186/1742-4682-6-11 (PMC2709892; doi:10.1186/1742-4682-6-11)
Supplement: Additional file 4 — Reproduction numbers. This document shows how equation 1 can be derived. [file 1742-4682-6-11-S4.pdf]

Additional file 4 – Reproduction numbers

Definitions

In the literature, there are several definitions of the basic reproduction number and also many methods to calculate it. As this wealth of similar, but not identical definitions is prone to cause misunderstandings, we provide here a definition for our use of both the term *basic reproduction number* and the term *reproduction number*.

For the basic reproduction number R_0 we rely on the classical definition that R_0 is the expected (≈ average) number of secondary cases that are induced by one infector introduced into a completely susceptible population. This classical definition of R_0 is no longer meaningful if the properties of the individuals are highly heterogeneous (e.g. high dispersion in the degree distribution). However, as the populations used in our simulation runs are rather homogeneous with respect to their network properties, the use of the easily comprehensible classical definition is reasonable.

With reproduction number R we describe the expected number of secondary cases caused by an infector introduced in any (clearly defined) population. The requirement of a completely susceptible population is relaxed, which means that R is the generalization of R_0.

Reproduction numbers for models without clustering

For the random mixing model, the basic reproduction number $R_{0,ran}$ is given by

$$R_{0,ran} \cong \beta \cdot n \cdot \tau \qquad (1)$$

and the reproduction number in general, R_{ran}, is given by

$$R_{ran} \cong \beta \cdot \frac{S}{N} n \cdot \tau \qquad (2)$$

where β denotes the per day transmission probability of one susceptible-infectious pair. n stands for the number of daily contacts, τ for the infectious period (in days), S for the total number of susceptible individuals in the entire population and N for the total population size. Both relations are only approximate as they assume S/N to be constant during τ. $R_{ran} \to R_{0,ran}$ for $S/N \to 1$.

In case of daily repeating contacts R_{rep} can be derived by the following line of arguments: The probability that a given susceptible person becomes infected is the complementary probability of the event that this person becomes not infected. If an infector meets a susceptible individual on τ days, the probability that no transmission occurs is

$$p_{IS \to RS} = (1-\beta)^{\tau}. \qquad (3)$$

Accordingly, the complementary probability that I infects S is

$$p_{IS \to RI} = 1 - (1-\beta)^{\tau} . \tag{4}$$

The reproduction number stands for the expected value of secondary cases generated by an infector in a given situation. R_{rep} is approximately given by the number of susceptible individuals in the infector's set of contacts times the probability of infection during τ . If contacts repeat daily, but are randomly distributed over the whole population, the expected number of susceptible individuals in the set of contacts is

$$s = \frac{S}{N}(n-1) \tag{5}$$

In contrast to the case of pure random mixing, the contact that originally infected the infector in consideration is permanently in the set of the infector's contacts. Therefore the set of susceptible contacts has to be smaller than the total set of contacts by at least one individual.

R_{rep} is approximately the product of equation 4 and equation 5:

$$R_{rep} \cong \frac{S}{N}(n-1) \cdot \left[1-(1-\beta)^{\tau}\right] \tag{6}$$

As the reproduction number turns into the basic reproduction number for $S/N \to 1$, $R_{0,rep}$ is given by

$$R_{0,rep} \cong (n-1) \cdot \left[1-(1-\beta)^{\tau}\right] \tag{7}$$

Both equation 6 and equation 7 are only approximate relations as they assume S/N to be constant during τ .
